# Sensor-Derived Mechanism-Informed Prediction of Section-Level Residual Profile Error in Robotic Blade-Edge Finishing

**DOI:** 10.3390/s26123799

**Published:** 2026-06-15

**Authors:** Zhuohang Gao, Xi Zeng, Zhenyu Cai, Cong Wen

**Affiliations:** College of Mechanical Engineering, Zhejiang University of Technology, 288 Liuhe Road, Xihu District, Hangzhou 310023, China; zhuohanggao713@gmail.com (Z.G.); 221123020262@zjut.edu.cn (Z.C.); congwen@zjut.edu.cn (C.W.)

**Keywords:** robotic blade-edge finishing, CMM metrology, mechanism-informed learning, Gaussian process regression, residual profile-error prediction

## Abstract

Robotic belt finishing of turbine-blade edges is difficult to control because local edge radius, contact compliance, and the incoming profile state jointly affect the final residual profile error. This study develops a sensor-derived, mechanism-informed framework for predicting section-level root-mean-square (RMS) residual profile error. Online force measurements, robot and process records, CAD-derived edge geometry, and coordinate measuring machine (CMM) profiles are converted into interpretable section-level descriptors. Three coupled descriptors are introduced to represent the load-to-radius ratio, the force–radius-mismatch interaction, and the normalized radius mismatch. Four Gaussian process regression (GPR) configurations, a training-mean predictor, and a ridge-regression baseline are evaluated using a grouped leave-one-blade-out protocol on eight blades and 80 measured sections. The proposed descriptors show clear predictive value under blade-wise evaluation. Ridge-B3 achieves the best deterministic accuracy, with RMSE = 1.0285 µm and R^2^ = 0.7759. The predefined GPR-B3 model does not provide the lowest point-prediction error, but it provides predictive intervals and descriptor-attribution information. These results indicate that descriptor construction is the primary source of deterministic accuracy, whereas GPR serves as an uncertainty-aware modeling layer for risk-aware blade-edge quality assessment.

## 1. Introduction

Steam turbine blades are typical freeform components with thin wall structures, and their geometric accuracy and surface integrity directly affect aerodynamic performance and structural reliability. Robotic belt grinding has been widely investigated for blade finishing because it combines the motion flexibility of industrial robots with the compliant contact behavior of abrasive-belt machining [1,2]. This process is particularly suitable for complex curved surfaces, where the contact wheel and abrasive belt can better adapt to local geometric variations than rigid grinding tools [3,4]. Recent studies have further demonstrated the potential of robotic belt finishing for improving machining consistency and automation in blade manufacturing [5]. Nevertheless, the leading and trailing edges remain difficult to control. Their small local radii, rapid geometric transitions, and limited structural support make local material removal highly sensitive to contact variation, system compliance, and geometric mismatch.

Considerable efforts have been made to understand and control robotic blade grinding. Material removal mechanisms and equivalent grinding models have been developed to describe the relationship between contact conditions and local removal depth [1,6]. Force control and compliant machining strategies have also been proposed to stabilize the interactions among the robot, abrasive belt, and blade surface [7,8]. In addition, tool path planning and pose optimization have been used to improve accessibility and contact consistency during blade finishing [9,10]. Beyond robotic machining, sensor-derived state descriptors have also been used in robot planning and navigation studies to improve decision-making under uncertain operating conditions [11,12]. These studies support the general value of state-aware descriptor construction, but they do not address section-level residual profile-error prediction in compliant robotic finishing. For profile accuracy improvement, compensation methods have been proposed to reduce deterministic machining errors and improve the final blade geometry [10,13,14,15]. These studies have improved the understanding of process mechanisms and compensation design. However, most existing approaches still rely mainly on deterministic error estimates and provide limited uncertainty information. For blade-edge finishing with limited data, this limitation may reduce prediction robustness when process variation, local anomalies, or geometric mismatch occur.

Gaussian process regression (GPR) is suitable for limited-sample process modeling because it provides both predictive means and uncertainty estimates. Gaussian process-based and Bayesian regression models have been used for machining-error prediction, process monitoring, and compensation-related modeling under data-limited conditions [16,17,18,19]. Recent physics-informed [20,21] or mechanism-assisted [22,23] learning studies further show that embedding process descriptors into statistical models can improve interpretability and generalization [24,25]. However, existing applications mainly address tool wear evolution, scalar removal responses, global machining-error prediction, or dense surface-deviation modeling. Section-level prediction of residual blade-edge profile error in robotic belt finishing remains insufficiently studied, particularly when blade-level samples are limited and heterogeneous sensing and metrology data must be integrated.

In the finishing of blade leading and trailing edges, the final sectional profile error is affected by contact load, local edge radius, radius mismatch between the measured incoming edge profile and the nominal CAD edge profile, robot pose, and spanwise location. These factors interact through the compliant contact among the blade, robot, and abrasive-belt wheel, making nominal process parameters insufficient for accurate prediction at the sectional level. Moreover, because multiple measured sections are obtained from the same physical blade, model validation must preserve blade grouping to avoid overly optimistic estimates of generalization performance.

To address this need, this study develops a sensor-derived, mechanism-informed prediction framework for section-level RMS residual profile error in robotic blade-edge finishing. The framework transforms online force measurements, robot and process records, CAD-derived local geometry, and CMM-based sectional metrology into physically interpretable descriptors. Coupled descriptors are constructed to encode interactions among contact load, local edge radius, and incoming-to-nominal radius mismatch. GPR is used to provide uncertainty-aware prediction, whereas deterministic baselines are included to evaluate the predictive value of the descriptor system independently from the probabilistic model class. The framework therefore provides an interpretable route for cross-blade residual-error assessment under limited blade-level data.

The main contributions are summarized as follows:

1. A section-level formulation is established for predicting residual profile error in robotic blade-edge finishing by linking online force sensing, robot records, process records, CAD geometry, and CMM sectional metrology.
2. A descriptor system derived from sensor data and informed by the deformation mechanism is constructed from measurable variables associated with contact load, local edge geometry, incoming profile state, edge type, and spanwise location. Low-order coupled descriptors are further introduced to represent the interaction effects among contact load, local geometry, and local edge-radius mismatch without requiring a separately calibrated analytical prior mean.
3. A grouped leave-one-blade-out evaluation protocol is adopted to reduce data leakage between training and test samples. In this protocol, all measured sections from the same physical blade are kept in the same fold, so that generalization to unseen blades can be assessed more conservatively.
4. Deterministic baselines and probabilistic Gaussian process regression configurations are evaluated under the same grouped protocol. This comparison separates the predictive value of the proposed descriptors from the effects of different model families. The full descriptor ridge-regression baseline is used to assess deterministic predictive capability, whereas the Gaussian process regression configurations are used to provide probabilistic prediction and feature attribution interpretation.

The remainder of this paper is organized as follows. Section 2 formulates the edge finishing problem and analyzes the coupled deformation mechanism. Section 3 presents the mechanism-informed GPR framework. Section 4 describes the experimental setup, variable acquisition, measurement strategy, and evaluation protocol. Section 5 reports the comparative results, and Section 6 concludes the paper.

## 2. Coupled Deformation Mechanism Analysis

Residual profile generation in robotic blade-edge finishing is formulated as a coupled deformation process. This section defines the pointwise profile deviation obtained from sectional metrology, establishes the deformation chain linking nominal engagement to actual material removal through workpiece retreat, robot end deflection, and contact-wheel deformation, and introduces an equivalent compliance representation for constructing the descriptors used in the subsequent regression model.

### 2.1. Local Sectional Coordinate System and Definition of Pointwise Profile Deviation

Let s∈[0,L] denote the local arc length coordinate of the edge profile extracted from a measured spanwise section. For notational simplicity, the section index is omitted in this subsection. The reference sectional profile is denoted by $p_{\mathrm{ref}}\left(s_{j}\right)$, the measured profile before grinding by $p_{0}\left(s_{j}\right)$, and the measured profile after grinding by $p_{f}\left(s_{j}\right)$. The local unit normal vector defined on the reference profile is denoted by $n(s_{j})$. All measured contours are first registered to the nominal section. The sampled points used for deviation calculation are then mapped onto the reference arc length coordinate through interpolation and normal projection.

**Figure 1:**
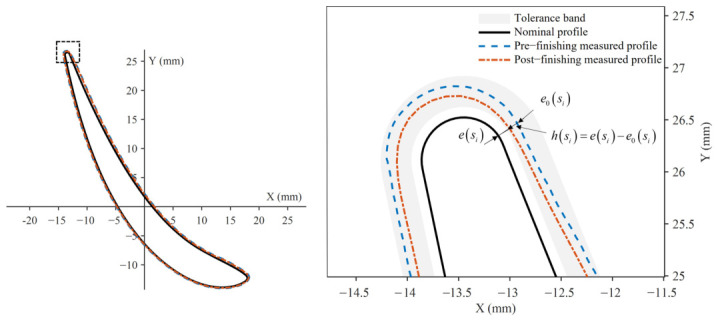
Definition of pointwise profile deviation on a measured blade-edge section.

To clarify the geometric definition of sectional profile deviation, Figure 1 illustrates the local coordinate system used in this study. The figure shows the reference profile, the measured profiles before and after grinding, the local outward normal direction, and the sign convention for sectional profile deviation and material removal.

Based on the normal projection of the measured profile onto the reference profile, the initial pointwise profile deviation before grinding is defined as

(1) $$e_{0}\left(s_{j}\right)=n^{⊤}\left(s_{j}\right)\left[p_{0}\left(s_{j}\right)-p_{\mathrm{ref}}\left(s_{j}\right)\right]$$

and the residual pointwise profile deviation after grinding is defined as

(2) $$e\left(s_{j}\right)=n^{⊤}\left(s_{j}\right)\left[p_{f}\left(s_{j}\right)-p_{\mathrm{ref}}\left(s_{j}\right)\right]$$

Here, a positive value indicates residual stock along the outward normal direction, whereas a negative value indicates local overcutting.

Accordingly, the local material removal at the sampled position is written as

(3) $$h\left(s_{j}\right)=e_{0}\left(s_{j}\right)-e\left(s_{j}\right)$$

Equations (1)–(3) define the pointwise profile deviation and local removal fields on each measured blade-edge section. These fields provide the basis for sectional registration, geometric feature extraction, and response construction. The regression response used in this study is obtained by aggregating the residual pointwise deviation over each section into a section-level RMS residual profile error.

### 2.2. Residual Edge Profile-Error Generation Under Coupled Deformation

In robotic belt grinding, the residual sectional profile is determined by the actual local removal generated under the deformed contact state. The residual pointwise profile deviation after grinding is expressed as

(4) $$e\left(s\right)=e_{0}\left(s\right)-h^{\mathrm{act}}\left(s\right)+\varepsilon_{m}\left(s\right)$$

where $h^{\mathrm{act}}\left(s\right)$ denotes the actual local removal depth, and $\varepsilon_{m}\left(s\right)$ represents measurement noise and unmodeled process disturbances.

**Figure 2:**
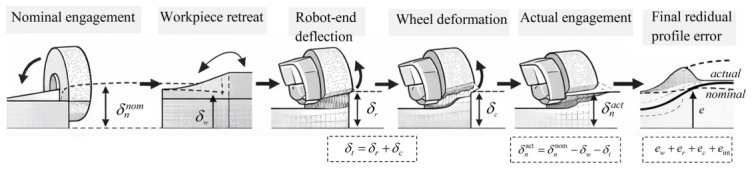
Residual edge profile-error generation under coupled deformation in robotic belt grinding.

The actual local removal depth is governed by the actual normal engagement and the local contact condition:

(5) $$h^{\mathrm{act}}\left(s\right)=g\;\left(\delta_{n}^{\mathrm{act}}\left(s\right),\;{\bar{F}}_{n},\;v_{f},\;v_{b},\;\Delta R,\;\ldots \right)$$

where g(·) denotes the removal relation under coupled deformation, $\delta_{n}^{\mathrm{act}}\left(s\right)$ is the actual normal engagement, ${\bar{F}}_{n}$ is the steady-state mean normal force, $v_{f}$ is the feed speed, $v_{b}$ is the belt speed, and ΔR is the local radius mismatch. In this study, ΔR describes the radius difference between the fitted effective edge radius of the incoming measured profile and the nominal local edge radius. The ellipsis denotes additional contact and process variables not explicitly included in the section-based regression formulation. The residual edge profile-error generation mechanism under coupled deformation is illustrated in Figure 2.

The actual normal engagement is written as

(6) $$\delta_{n}^{\mathrm{act}}=\delta_{n}^{\mathrm{nom}}-\delta_{w}-\delta_{r}-\delta_{c}+\delta_{\mathrm{int}}$$

where $\delta_{n}^{\mathrm{nom}}$ and $\delta_{n}^{\mathrm{act}}$ denote the nominal and actual normal engagement, respectively. The terms $\delta_{w}$, $\delta_{r}$, and $\delta_{c}$ denote the normal retreat of the workpiece, the normal deflection at the robot end, and the wheel contact deformation. These deformation terms are defined as positive when they reduce the nominal normal engagement. The term $\delta_{\mathrm{int}}$ is a signed correction term for residual coupling among the deformation components; a positive value increases the actual engagement, whereas a negative value decreases it.

Equation (6) gives the deformation balance that links nominal engagement to actual engagement. Together with Equation (5), it provides the mechanism basis for constructing the compliance representation and coupled descriptors in the following subsection.

### 2.3. Section-Level Descriptor Construction from the Deformation Mechanism

The deformation quantities introduced above are expressed in equivalent compliance form to construct the descriptor block used in the regression model. At edge location *s*, the equivalent workpiece retreat, robot end deflection, and wheel contact deformation are defined as

(7) $$\delta_{w}^{0}\left(s\right)=\frac{{\bar{F}}_{n}}{k_{w}^{0}\left(s\right)}$$

(8) $$k_{r}^{0}\left(q,\theta ,s\right)={\left[n^{⊤}\left(s\right)\;J_{v}\left(q,\theta \right)\;K_{q}^{-1}\;J_{v}^{⊤}\left(q,\theta \right)\;n\left(s\right)\right]}^{-1}$$

(9) $$\delta_{r}^{0}\left(q,\theta ,s\right)=\frac{{\bar{F}}_{n}}{k_{r}^{0}\left(q,\theta ,s\right)}$$

and

(10) $$\delta_{c}^{0}\left(p,\Delta R\right)=\frac{{\bar{F}}_{n}}{k_{c}^{0}\left(p,\Delta R\right)}$$

Here, ${\bar{F}}_{n}$ denotes the steady-state mean normal force, $k_{w}^{0}\left(s\right)$ denotes the nominal local support stiffness of the blade edge, and $k_{r}^{0}\left(q,\theta ,s\right)$ denotes the equivalent normal stiffness of the robot along the local normal direction. The vector q denotes the joint configuration, θ denotes the tool orientation angle, $J_{v}\left(q,\theta \right)$ denotes the translational Jacobian at the contact point, and $K_{q}$ denotes the joint stiffness matrix. The term $k_{c}^{0}\left(p,\Delta R\right)$ denotes the effective stiffness of the wheel contact system, where *p* and ΔR represent the wheel process state and the local radius mismatch, respectively.

The first-order equivalent deformation is written as

(11) $$\delta_{\mathrm{def}}^{0}=\delta_{w}^{0}+\delta_{r}^{0}+\delta_{c}^{0}$$

Introducing the equivalent compliances

(12) $$C_{w}\left(s\right)=\frac{1}{k_{w}^{0}\left(s\right)},\;C_{r}\left(q,\theta ,s\right)=\frac{1}{k_{r}^{0}\left(q,\theta ,s\right)},\;C_{c}\left(p,\Delta R\right)=\frac{1}{k_{c}^{0}\left(p,\Delta R\right)}$$

where, $C_{w}\left(s\right)$, $C_{r}\left(q,\theta ,s\right)$, and $C_{c}\left(p,\Delta R\right)$ are the corresponding equivalent normal compliances of the workpiece, robot, and wheel contact system, respectively. Equation (11) becomes

(13) $$\delta_{\mathrm{def}}^{0}\left(s\right)={\bar{F}}_{n}\left[C_{w}\left(s\right)+C_{r}\left(q,\theta ,s\right)+C_{c}\left(p,\Delta R\right)\right]$$

This equation gives the local force and compliance representation of the deformation chain. The normal load enters the deformation expression through the workpiece compliance, robot compliance, and wheel contact compliance.

For the workpiece contribution, the leading section-level dependence of the local compliance is represented by the local curvature. With $\kappa_{l}\left(s\right)$ denoting the local edge curvature,

(14) $$C_{w}\left(s\right)\approx a_{w0}+a_{w1}\kappa_{l}\left(s\right)\;\kappa_{l}\left(s\right)\approx \frac{1}{R_{l}}$$

Substitution into Equation (7) gives

(15) $$\begin{array}{cc} \delta_{w}^{0}\left(s\right)\; & ={\bar{F}}_{n}C_{w}\left(s\right)\approx a_{w0}{\bar{F}}_{n}+a_{w1}\frac{{\bar{F}}_{n}}{R_{l}} \end{array}$$

The term ${\bar{F}}_{n}$ belongs to the basic load descriptor set, while the curvature-scaled load component defines the first coupled descriptor,

(16) $$\phi_{1}=\frac{{\bar{F}}_{n}}{R_{l}}$$

For the robot contribution, $C_{r}\left(q,\theta ,s\right)$ is determined by the joint configuration, tool orientation, and local normal direction. In the section-level descriptor system, this dependence is represented through the measured tool orientation and section context variables. Accordingly, no additional robot stiffness interaction variable is added to the coupled descriptor block defined in this subsection.

For the wheel contact contribution, the local radius mismatch is defined as

(17) $$\Delta R=R_{\mathrm{eff}}-R_{l}$$

where $R_{l}$ is the nominal local edge radius extracted from the CAD section, and $R_{\mathrm{eff}}$ denotes the effective local edge radius fitted from the registered pre-finishing measured profile within the same evaluation window. Thus, ΔR represents the incoming edge-radius deviation relative to the nominal CAD geometry. It is not the physical contact-wheel radius; rather, it is used as a measurable descriptor of local wheel–edge geometric compatibility.

A first-order expansion of the wheel contact compliance with respect to ΔR at a representative operating point $\Delta R_{*}$ gives

(18) $$C_{c}\left(p,\Delta R\right)\approx C_{c}\left(p,\Delta R_{*}\right)+{\left.\frac{\partial C_{c}}{\partial \Delta R}\right|}_{\Delta R_{*}}\left(\Delta R-\Delta R_{*}\right)$$

Equivalently,

(19) $$C_{c}\left(p,\Delta R\right)\approx b_{c0}\left(p\right)+b_{c1}\left(p\right)\Delta R$$

where $b_{c0}\left(p\right)$ and $b_{c1}\left(p\right)$ collect the constant and derivative terms in the local expansion. The corresponding wheel contact deformation is

(20) $$\begin{array}{cc} \delta_{c}^{0} & ={\bar{F}}_{n}C_{c}\left(p,\Delta R\right)\approx b_{c0}\left(p\right){\bar{F}}_{n}+b_{c1}\left(p\right){\bar{F}}_{n}\Delta R \end{array}$$

The first term is associated with the force descriptor and wheel process state. The second term defines the force-weighted radius mismatch descriptor,

(21) $$\phi_{2}={\bar{F}}_{n}\Delta R$$

The radius mismatch is also normalized by the local edge scale. Let $\kappa_{\mathrm{eff}}=1/R_{\mathrm{eff}}$ denote the effective contact curvature corresponding to $R_{\mathrm{eff}}$. Since

(22) $$R_{\mathrm{eff}}=R_{l}+\Delta R$$

the relative curvature mismatch is expressed as

(23) $$\frac{\kappa_{l}-\kappa_{\mathrm{eff}}}{\kappa_{l}}=1-\frac{R_{l}}{R_{l}+\Delta R}=\frac{\Delta R}{R_{l}+\Delta R}=\frac{\Delta R/R_{l}}{1+\Delta R/R_{l}}$$

The ratio $\Delta R/R_{l}$ is therefore retained as the normalized radius mismatch descriptor,

(24) $$\phi_{3}=\frac{\Delta R}{R_{l}}$$

For the *i*th measured section, the coupled descriptor block is defined as

(25) $$\phi_{i}={\left[\phi_{1,i},\phi_{2,i},\phi_{3,i}\right]}^{⊤}={\left[\frac{{\bar{F}}_{n,i}}{R_{l,i}},{\bar{F}}_{n,i}\Delta R_{i},\frac{\Delta R_{i}}{R_{l,i}}\right]}^{⊤}$$

The three components correspond to the curvature-scaled load term, the force-weighted radius mismatch term, and the normalized radius mismatch term, respectively. The coefficients in the compliance expansions are not introduced as independent inputs; their effects are represented by the regression mapping after descriptor normalization. Thus, Equation (25) defines the mechanism-related interaction block appended to the basic section-level descriptor vector in the prediction model.

## 3. Section-Level Mechanism-Informed Prediction Framework

**Figure 3:**
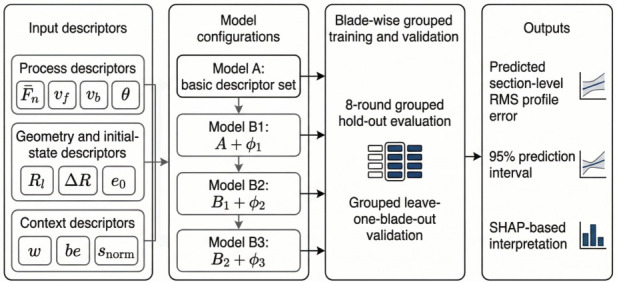
Overall workflow of the section-level mechanism-informed prediction framework.

The prediction framework maps section-level process, geometry, force sensing, and metrology variables to the RMS residual profile error of measured blade-edge sections. The coupled descriptor block derived from the deformation mechanism in Section 2 is appended progressively to the basic descriptor vector. Four Gaussian process regression configurations are evaluated together with deterministic references under the same blade-wise grouped protocol. The overall workflow is shown in Figure 3. The blocks represent the major stages of descriptor construction, model training, and performance evaluation, and the connecting lines indicate the data flow and the progressive addition of the coupled descriptor block.

### 3.1. Descriptor Set and Model Configurations

For the *i*th measured blade-edge section, the regression response is defined as the section-level RMS residual profile error,

(26) $$y_{i}={\left(\frac{1}{m_{i}}\sum_{j=1}^{m_{i}}e^{2}\left(s_{ij}\right)\right)}^{1/2}$$

where $m_{i}$ is the number of sampled points on section *i*, and $s_{ij}$ denotes the *j*th sampled position. The corresponding incoming profile state descriptor is

(27) $$E_{0,i}={\left(\frac{1}{m_{i}}\sum_{j=1}^{m_{i}}e_{0}^{2}\left(s_{ij}\right)\right)}^{1/2}$$

The pointwise deviation fields are used for registration, geometric feature extraction, and response construction. The regression model is trained on section-level samples rather than on individual contour points.

The basic descriptor vector of Model A is

(28) $$x_{i}^{\left(A\right)}={\left[{\bar{F}}_{n,i},v_{f,i},v_{b,i},\theta_{i},R_{l,i},\Delta R_{i},E_{0,i},b_{e,i},s_{\mathrm{norm},i}\right]}^{⊤}$$

where ${\bar{F}}_{n,i}$ is the steady-state mean normal force, $v_{f,i}$ is the feed speed, $v_{b,i}$ is the belt speed, $\theta_{i}$ is the tool orientation angle, $R_{l,i}$ is the representative local edge radius, $\Delta R_{i}$ is the radius mismatch descriptor, $E_{0,i}$ is the incoming profile state descriptor, $b_{e,i}$ is the edge-type indicator, and $s_{\mathrm{norm},i}$ is the normalized spanwise coordinate. The edge type indicator is

(29) $$b_{e,i}=\left\{\begin{array}{cc} 0, & \mathrm{leading}\;\mathrm{edge}\\ 1, & \mathrm{trailing}\;\mathrm{edge} \end{array}\right.$$

The pass-state record $p_{i}$ describes the chronological process context of the wheel–belt system. Because $p_{i}$ is a coarse ordinal indicator rather than a calibrated section-level measurement of belt wear or wheel compliance, it is retained for mechanism interpretation but is not used as an explicit input in the main regression implementation.

The coupled descriptor block $\phi_{i}={\left[\phi_{1,i},\phi_{2,i},\phi_{3,i}\right]}^{⊤}$ is defined in Equation (25). The four descriptor configurations used in the regression models are summarized in Table 1.

Model B3 denotes the full descriptor configuration considered in this study. The model hierarchy is fixed before grouped evaluation.

Before model fitting, continuous descriptors are normalized within each training fold. In the *r*th grouped round, the normalized value of the *k*th descriptor for Model *M* is

(30) $${\tilde{x}}_{i,k}^{\left(M,r\right)}=\frac{x_{i,k}^{\left(M\right)}-x_{k,\min}^{\left(M,r\right)}}{x_{k,\max}^{\left(M,r\right)}-x_{k,\min}^{\left(M,r\right)}}\;M\in \left\{A,B1,B2,B3\right\}$$

where $x_{k,\min}^{\left(M,r\right)}$ and $x_{k,\max}^{\left(M,r\right)}$ are computed only from the training subset of round *r*. The binary edge indicator is not normalized. If a continuous descriptor has zero range within a training subset, it is left unscaled in that round. The same training fold normalization constants are applied to the corresponding test blade without clipping. Therefore, normalized test values may fall outside [0,1] when the held-out blade contains descriptor values outside the training range.

### 3.2. Gaussian Process Regression and Deterministic References

For Model M∈{A,B1,B2,B3} in grouped round *r*, the GPR model was fitted using only the training subset of that round. The continuous input descriptors were first normalized using the training-fold statistics, and the same normalization constants were applied to the corresponding held-out blade without clipping. The response was kept on the original micrometer scale so that the predicted mean and predictive interval could be interpreted directly as a section-level RMS residual profile error.

For a training section $i\in I_{\mathrm{tr}}^{\left(r\right)}$, the model is written as

(31) $$y_{i}=\beta_{0}^{\left(M,r\right)}+f^{\left(M,r\right)}\left({\tilde{x}}_{i}^{\left(M,r\right)}\right)+\varepsilon_{i}^{\left(M,r\right)}$$

where $\beta_{0}^{\left(M,r\right)}$ is a constant basis term estimated from the training subset,

(32) $$\varepsilon_{i}^{\left(M,r\right)}∼N\left(0,{\left(\sigma_{n}^{\left(M,r\right)}\right)}^{2}\right)$$

and

(33) $$f^{\left(M,r\right)}\left(\cdot \right)∼\mathrm{GP}\left(0,k^{\left(M,r\right)}\left(\cdot ,\cdot \right)\right)$$

A Matérn 5/2 kernel with automatic relevance determination was used for the predefined main GPR configurations:

(34) $$k^{\left(M,r\right)}\left(\tilde{x},{\tilde{x}}^{\prime }\right)={\left(\sigma_{f}^{\left(M,r\right)}\right)}^{2}\left[1+\sqrt{5}\rho^{\left(M,r\right)}+\frac{5}{3}{\left(\rho^{\left(M,r\right)}\right)}^{2}\right]\exp\left(-\sqrt{5}\rho^{\left(M,r\right)}\right)$$

with

(35) $$\rho^{\left(M,r\right)}={\left[\sum_{k=1}^{d_{M}}\frac{{\left({\tilde{x}}_{k}-{\tilde{x}}_{k}^{\prime }\right)}^{2}}{{\left(\ell_{k}^{\left(M,r\right)}\right)}^{2}}\right]}^{1/2}$$

Here, $d_{M}$ is the descriptor dimension of Model *M*, $\sigma_{f}^{\left(M,r\right)}$ is the signal standard deviation, and $\ell_{k}^{\left(M,r\right)}$ is the characteristic length scale of the *k*th descriptor in the normalized input space.

The kernel hyperparameter vector is

(36) $$\psi^{\left(M,r\right)}={\left[\sigma_{f}^{\left(M,r\right)},\ell_{1}^{\left(M,r\right)},\ldots ,\ell_{d_{M}}^{\left(M,r\right)},\sigma_{n}^{\left(M,r\right)}\right]}^{⊤}$$

The constant basis term $\beta_{0}^{\left(M,r\right)}$ was estimated from the same training subset but was not treated as a kernel capacity parameter.

For each model configuration and each outer fold, the kernel hyperparameters and the constant basis term were estimated by minimizing the negative log marginal likelihood on the training subset:

(37) $$\psi^{\left(M,r\right)}=\arg\min_{\psi }L_{\mathrm{NLML}}\left(\psi ;{\left\{{\tilde{x}}_{i}^{\left(M,r\right)},y_{i}\right\}}_{i\in I_{\mathrm{tr}}^{\left(r\right)}}\right)$$

No held-out blade samples were used during descriptor normalization, model fitting, or hyperparameter learning.

Let $K_{f,\mathrm{tr}}^{\left(M,r\right)}$ denote the latent-function covariance matrix computed from Equation (34) on the training subset. The covariance matrix used for marginal-likelihood optimization and posterior prediction is

(38) $$K_{y,\mathrm{tr}}^{\left(M,r\right)}=K_{f,\mathrm{tr}}^{\left(M,r\right)}+{\left(\sigma_{n}^{\left(M,r\right)}\right)}^{2}I$$

Numerical conditioning was handled as part of the exact GPR fitting procedure. Any numerical stabilization used for matrix computation was not interpreted as an additional physical noise source.

For a held-out test section with normalized descriptor vector ${\tilde{x}}_{*}^{\left(M,r\right)}$, let

(39) $$k_{*}^{\left(M,r\right)}={\left[k^{\left(M,r\right)}\left({\tilde{x}}_{*}^{\left(M,r\right)},{\tilde{x}}_{j}^{\left(M,r\right)}\right)\right]}_{j\in I_{\mathrm{tr}}^{\left(r\right)}}$$

The posterior predictive mean on the original micrometer scale is

(40) $${\hat{\mu }}_{y,*}^{\left(M,r\right)}=\beta_{0}^{\left(M,r\right)}+{\left(k_{*}^{\left(M,r\right)}\right)}^{⊤}{\left(K_{y,\mathrm{tr}}^{\left(M,r\right)}\right)}^{-1}\left(y_{\mathrm{tr}}^{\left(r\right)}-\beta_{0}^{\left(M,r\right)}1\right)$$

and the observed-response predictive variance is

(41) $${\left({\hat{\sigma }}_{y,*}^{\left(M,r\right)}\right)}^{2}=k^{\left(M,r\right)}\left({\tilde{x}}_{*}^{\left(M,r\right)},{\tilde{x}}_{*}^{\left(M,r\right)}\right)-{\left(k_{*}^{\left(M,r\right)}\right)}^{⊤}{\left(K_{y,\mathrm{tr}}^{\left(M,r\right)}\right)}^{-1}k_{*}^{\left(M,r\right)}+{\left(\sigma_{n}^{\left(M,r\right)}\right)}^{2}$$

The last term in Equation (41) includes the fitted observation-noise contribution, so the resulting standard deviation corresponds to the predictive distribution of the measured section-level response rather than only the latent function.

The nominal 95% predictive interval for the observed section-level RMS residual profile error is therefore

(42) $$\left[{\hat{\mu }}_{y,*}^{\left(M,r\right)}-1.96{\hat{\sigma }}_{y,*}^{\left(M,r\right)},\;{\hat{\mu }}_{y,*}^{\left(M,r\right)}+1.96{\hat{\sigma }}_{y,*}^{\left(M,r\right)}\right]$$

Because the response is a non-negative RMS quantity and the dataset contains only eight independent blades, this Gaussian interval is treated as a nominal predictive interval rather than as a complete metrological uncertainty bound.

After model fitting, the optimized ARD length scales were retained as diagnostic quantities. A large optimized length scale indicates that the fitted covariance function varies slowly along the corresponding descriptor direction, whereas a smaller length scale indicates stronger local sensitivity. These length-scale diagnostics were used only for interpreting the fitted GPR behavior and were not used for test-set-based model selection.

Two deterministic references were evaluated under the same grouped protocol. The training-mean predictor assigns the mean response of the training subset to all test sections in the held-out blade:

(43) $${\hat{y}}_{i,\mathrm{mean}}^{\left(r\right)}={\bar{y}}_{\mathrm{tr}}^{\left(r\right)}=\frac{1}{|I_{\mathrm{tr}}^{\left(r\right)}|}\sum_{j\in I_{\mathrm{tr}}^{\left(r\right)}}y_{j},\;i\in I_{\mathrm{te}}^{\left(r\right)}$$

The ridge-regression reference uses the full Model B3 descriptor vector and estimates

(44) $$\min_{\beta_{0},\beta }\sum_{i\in I_{\mathrm{tr}}^{\left(r\right)}}{\left[y_{i}-\beta_{0}-{\left({\tilde{x}}_{i}^{\left(B3,r\right)}\right)}^{⊤}\beta \right]}^{2}+\lambda {∥\beta ∥}_{2}^{2}$$

The regularization coefficient λ was selected within the training blades of each outer round by an inner grouped leave-one-blade-out procedure. Thus, Ridge-B3 uses the same full descriptor set as GPR-B3 but evaluates the deterministic predictive value of the descriptors under a regularized linear mapping.

### 3.3. Grouped Training and Prediction Protocol

A blade-wise grouped leave-one-blade-out protocol is adopted to evaluate cross-blade generalization. Let B={1,…,8} denote the set of physical blades, and let $I_{b}$ denote the index set of the ten measured edge sections from blade *b*. In the *r*th grouped round, blade $b_{r}$ is held out for testing, and the remaining blades are used for training:

(45) $$I_{\mathrm{te}}^{\left(r\right)}=I_{b_{r}},\;I_{\mathrm{tr}}^{\left(r\right)}=\underset{b\in B,\;b\neq b_{r}}{⋃}I_{b},\;r=1,\ldots ,8$$

Accordingly,

(46) $$\left|I_{\mathrm{tr}}^{\left(r\right)}\right|=70,\;\left|I_{\mathrm{te}}^{\left(r\right)}\right|=10,\;I_{\mathrm{tr}}^{\left(r\right)}\cap I_{\mathrm{te}}^{\left(r\right)}=⌀$$

All sections from the same physical blade are therefore assigned to the same subset. This design prevents information leakage caused by using sections from one blade simultaneously for training and testing.

For each outer round, all preprocessing and model-learning operations are performed using only $I_{\mathrm{tr}}^{\left(r\right)}$. The min–max normalization constants in Equation (30) are computed from the training subset and then applied to the corresponding held-out blade. The test descriptors are not clipped after normalization; values outside [0,1] are retained as fold-wise extrapolation cases. The number of such out-of-training-range descriptor values is reported with the prediction results.

For the GPR configurations M∈{A,B1,B2,B3}, the kernel hyperparameters and the constant basis term are estimated from the training subset of each round by marginal-likelihood optimization. The fitted model is then applied to the ten held-out sections of the test blade to obtain the predictive mean ${\hat{\mu }}_{y,i}^{\left(M\right)}$ and the observed-response predictive standard deviation ${\hat{\sigma }}_{y,i}^{\left(M\right)}$. For the deterministic references, the training-mean predictor and Ridge-B3 are also fitted only on $I_{\mathrm{tr}}^{\left(r\right)}$. The ridge regularization coefficient is selected within the training blades of the corresponding outer round by an inner grouped leave-one-blade-out procedure.

After all eight grouped rounds are completed, the held-out GPR prediction records are pooled as

(47) $$P_{\mathrm{GPR}}^{\left(M\right)}=\overset{8}{\underset{r=1}{⋃}}\left\{\left({\hat{\mu }}_{y,i}^{\left(M\right)},{\hat{\sigma }}_{y,i}^{\left(M\right)},y_{i},r\right)\;|\;i\in I_{\mathrm{te}}^{\left(r\right)}\right\}\;M\in \left\{A,B1,B2,B3\right\}$$

For the deterministic references, the same pooling operation is applied after omitting the predictive standard deviation. The pooled records contain 80 held-out section-level predictions. They are used for overall section-level performance assessment, while the fold index *r* is retained for held-out-blade-level paired comparison.

## 4. Experimental Setup and Evaluation Protocol

### 4.1. Experimental Platform and Blade Specimens

The robotic belt finishing experiments were carried out on an ABB IRB6700-2.6 industrial robot platform. Steam turbine blades made of 2Cr13 stainless steel, which are widely used in turbine generator units, were selected as the workpieces. The overall blade size is approximately 215mm×70mm.

**Figure 4:**
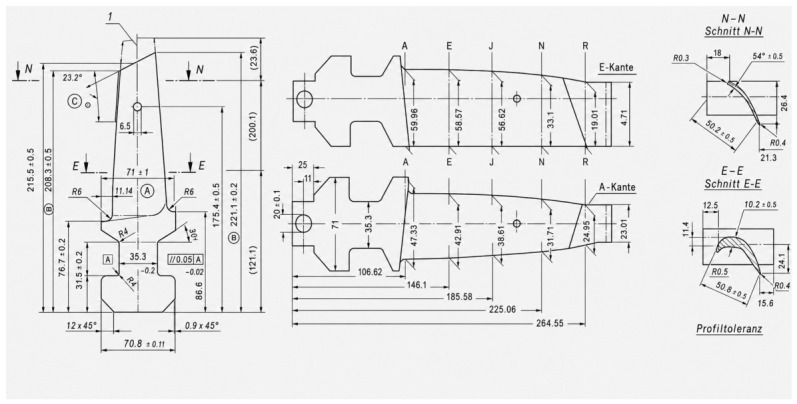
Drawing-defined blade geometry and edge-sectional profile inspection requirement of the turbine-blade specimen.

The drawing-defined blade geometry and edge-sectional inspection references are shown in Figure 4. The drawing identifies the inspected blade-edge regions, representative spanwise section marks, and local sectional geometry near the edge profiles. The main inspected edge fillet feature is specified as R0.3mm, and the edge-section inspection requirement is an allowable sectional RMS profile error of 0.2mm.

To monitor the interaction loads during finishing, a six-axis force/torque sensor (ATI OMEGA160, calibration model SI-1500-240 [26]) was mounted between the flange at the robot end and a pneumatic gripper. The blade was clamped by the pneumatic gripper and brought into contact with a fixed belt finishing device. During the finishing process, the sensor was used to measure the external normal contact force, tangential disturbance force, and the corresponding moments acting on the blade. After signal filtering, coordinate transformation, and gravity compensation, stable external contact-load information was obtained for subsequent process monitoring and model construction.

**Figure 5:**
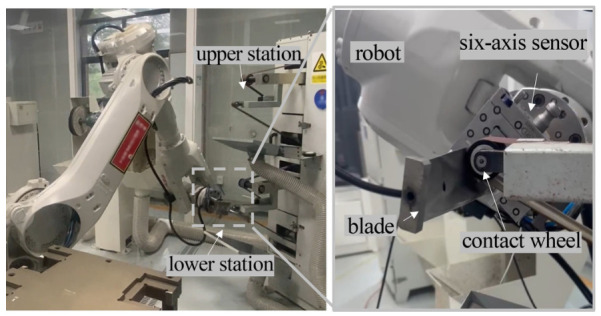
Experimental platform of the robotic belt finishing system.

The finishing unit was a dual station belt finishing device developed in-house, as shown in Figure 5. The upper station was mainly used for rough stock removal and was equipped with a 240-grit silicon carbide abrasive belt. The lower station was mainly used for semi-finishing and finishing and was equipped with a 600-grit silicon carbide abrasive belt to improve the local contact condition and enhance profile quality in the edge region. Both stations employed contact wheels with a diameter of 25mm and a width of 10mm as the compliant contact elements.

For all tested blades, the rough finishing, semi-finishing, and finishing procedures followed the prescribed process plan. The reserved stock before semi-finishing was the main blade-level condition used to generate different incoming profile states, whereas the station selection, abrasive-belt specification, contact-wheel geometry, and remaining process settings were maintained consistently. The common settings are summarized in Table 2.

The nominal stock allowance before semi-finishing is a planned blade-level process condition, whereas the variable directly available in the section-level dataset is the metrology-derived initial profile descriptor $E_{0,i}$. To characterize the realized incoming stock state of each blade, the measured blade-level initial state is defined as

(48) $$A_{b}^{\mathrm{meas}}=\frac{1}{10}\sum_{i\in b}E_{0,i}$$

where the summation is taken over the ten measured leading- and trailing-edge sections of blade *b*. Table 3 summarizes the incoming profile state obtained from the pre-finishing CMM profiles. Across the 80 measured edge sections, $E_{0}$ ranges from 7.19 μm to 17.38 μm, with a mean value of 13.72 μm and a standard deviation of 1.88 μm. The blade-level average $A_{b}^{\mathrm{meas}}$ describes the overall incoming state of each blade, while the section-level quantity $E_{0,i}$ is retained as the initial-state descriptor in the regression model.

### 4.2. Force Acquisition and Mechanism-Related Variable Extraction

The variables used in the present study were assembled from four information sources: online force sensing, robot controller records, grinding-unit settings, and offline sectional metrology. Let $f_{\mathrm{raw}}\left(t\right)$ denote the raw force signal measured by the sensor. After offset removal, gravity compensation, and coordinate transformation, the external contact-load vector can be written as

(49) $$f_{\mathrm{ext}}\left(t\right)=R_{s\to b}\left(t\right)\left[f_{\mathrm{raw}}\left(t\right)-f_{\mathrm{bias}}-f_{g}\left(t\right)\right]$$

where $R_{s\to b}\left(t\right)$ is the rotation matrix from the sensor frame to the blade/contact frame, $f_{\mathrm{bias}}$ is the sensor bias, and $f_{g}\left(t\right)$ is the gravity term associated with the end-effector and clamped workpiece.

Based on the local outward unit normal vector n(t) at the contact point, the instantaneous normal contact force is calculated as

(50) $$F_{n}\left(t\right)=n^{⊤}\left(t\right)f_{\mathrm{ext}}\left(t\right)$$

and the tangential disturbance component is written as

(51) $$F_{t}\left(t\right)={∥f_{\mathrm{ext}}\left(t\right)-F_{n}\left(t\right)n\left(t\right)∥}_{2}$$

For each grinding pass, the mean normal force over the steady-state contact interval $\left[t_{s},t_{e}\right]$ is defined as

(52) $${\bar{F}}_{n}=\frac{1}{t_{e}-t_{s}}\int_{t_{s}}^{t_{e}}F_{n}\left(t\right)\;dt$$

The steady-state mean normal force ${\bar{F}}_{n}$ was used as the load-related descriptor in the section-level predictor because it directly corresponds to the local normal indentation governing profile correction. The tangential force component $F_{t}$ and the moment channels were retained as auxiliary contact-state signals for identifying stable engagement and checking process consistency. Since the response variable $y_{i}$ is defined from the normal-direction residual profile deviation of each measured section, the regression input was restricted to descriptors with a direct section-level correspondence to this normal-profile response. In the absence of a separately identified friction or eccentric-contact model, $F_{t}$ and the moment channels were not reduced to additional scalar predictors in the main implementation.

The remaining process descriptors were obtained from the robot controller and the grinding-unit records. The feed speed $v_{f}$ and tool orientation angle θ were extracted from robot controller records, whereas the belt speed $v_{b}$ and pass-state record *p* were obtained from the grinding-unit process records. The pass-state record *p* provides chronological context for the wheel–belt state during the finishing sequence. In the implemented section-level predictor, *p* was retained for mechanism interpretation rather than encoded as a standalone regression input, because it records process order rather than a calibrated section-level measurement of belt wear or wheel compliance. Accordingly, the implemented predictor uses the measured normal force, process speeds, tool orientation, local geometry, initial profile state, edge identity, spanwise location, and the coupled descriptors defined in Section 3.1. The robot joint configuration $q_{i}$ was used only for stiffness-related physical interpretation.

The local radii used in the descriptor set were calculated from the CAD section and the registered CMM profile using the same evaluation window. The nominal local radius $R_{l,i}$ was obtained by arc fitting on the edge window of the nominal CAD section. The effective local radius $R_{\mathrm{eff},i}$ was obtained from the registered pre-finishing measured profile of section *i* by using the same arc-fitting window. Both radii describe the blade-edge geometry within the profile-evaluation window and should be distinguished from the contact-wheel size in the finishing unit.

The radius mismatch is defined as

(53) $$\Delta R_{i}=R_{\mathrm{eff},i}-R_{l,i}$$

Accordingly,

(54) $$R_{\mathrm{eff},i}=R_{l,i}+\Delta R_{i}$$

Across the 80 measured sections, $R_{l,i}$ ranges from 0.081 mm to 0.557 mm, with a mean of 0.318 mm and a standard deviation of 0.150 mm. The radius mismatch $\Delta R_{i}$ ranges from 0.005 mm to 0.142 mm, with a mean of 0.058 mm and a standard deviation of 0.038 mm. The resulting $R_{\mathrm{eff},i}$ ranges from 0.154 mm to 0.694 mm. Since $\Delta R_{i}$ is a difference between two fitted radii, it is reported as a radius mismatch instead of a curvature mismatch. Its numerical variation is affected by profile registration, the selected edge window, and arc fitting residuals; these effects are included in the metrology and model noise terms.

The stiffness quantities introduced in Section 2 were evaluated at the nominal level. The blade stiffness map $k_{w}^{0}\left(s\right)$ was obtained from ABAQUS loading simulations of the edge region under representative boundary conditions. The robot stiffness $k_{r}^{0}\left(q,\theta ,s\right)$ was calculated from the Jacobian-based stiffness model using nominal joint stiffness parameters and recorded joint angles. The wheel-compliance term $k_{c}^{0}\left(p,\Delta R\right)$ summarizes the influence of process state and radius mismatch on the contact condition. The superscript 0 indicates that these quantities are nominal estimates used for mechanism interpretation, not independently identified stiffness maps.

In this study, $k_{w}^{0}\left(s\right)$, $k_{r}^{0}\left(q,\theta ,s\right)$, and $k_{c}^{0}\left(p,\Delta R\right)$ are used to motivate the descriptor set and the coupled variables in Section 2 and Section 3. The regression model is trained on the measured process, geometry, and metrology descriptors, without introducing a separately calibrated analytical prior mean. Finally, the initial profile descriptor $E_{0,i}$ and the response $y_{i}$ are computed from the registered profiles before and after finishing according to Equations (27) and (26).

### 4.3. Sectional Profile Measurement and Dataset Construction

The sectional profiles of the blade-edge regions were measured using a Global S05.07.05 coordinate measuring machine. The measurement focused on the leading and trailing edges, where the small local radii and rapid curvature variation make the final profile sensitive to local removal error.

**Figure 6:**
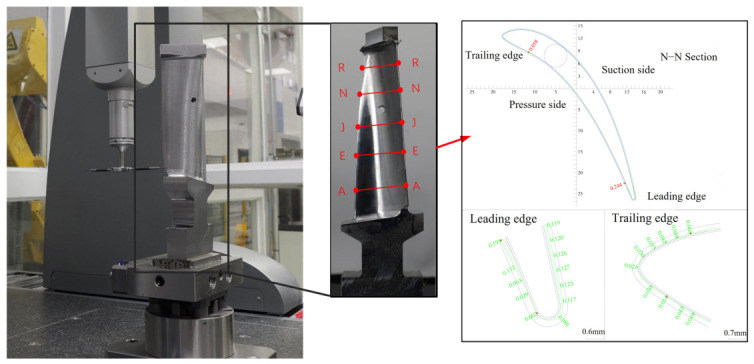
Sectional measurement strategy and section-level dataset construction.

As shown in Figure 6, the letters A, E, J, N, and R denote five selected spanwise measurement sections from the blade root/platform side to the blade tip side. Their locations were determined from the nominal sectional parameters of the blade CAD model, and their normalized spanwise coordinates were assigned as $s_{\mathrm{norm}}=0.1$, 0.3, 0.5, 0.7, and 0.9, respectively. For each blade, the leading-edge and trailing-edge profiles were measured at these five sections. Each blade therefore provided 10 local edge-section samples. Since eight blades were measured, the final dataset contained 80 section-level samples.

For each measured edge section, dense contour points were acquired within the local profile-evaluation window. The measured contour was registered to the corresponding nominal CAD section, and the pointwise profile-deviation field was then computed by normal projection according to the definitions in Section 2.

Although each sectional profile was measured as a dense contour, the regression dataset was constructed at the section level. The contour points were used for registration, geometric feature extraction, and response construction, but were not treated as independent regression samples. Following Equation (26), the scalar response $y_{i}$ of the *i*th measured edge section is the RMS residual profile error computed from the pointwise deviation field on that section. Similarly, the initial profile descriptor $E_{0,i}$ is computed according to Equation (27). In the 80 section-level samples, the Pearson correlation coefficient between $E_{0}$ and *y* is approximately 0.890, indicating that the incoming sectional profile state is a relevant descriptor for predicting the final residual error.

A repeatability check was further performed on five representative blade-edge sections covering both leading-edge and trailing-edge profiles. For the repeated probing check, each selected section was measured three times using the same probe path and probing speed as the main CMM measurement. For the repeated registration check, the same measured contour was registered five times to the nominal CAD section using perturbed initial poses. The combined check included repeated probing and registration, and the section-level RMS error was recomputed for each repeated result.

Table 4 summarizes the repeatability results for the selected sections. The standard deviation of the recomputed section-level RMS error remains below 0.50 μm in these checks. In the subsequent regression model, the residual contribution of metrology variation is included in the observation-noise term together with unmodeled process variation.

All 80 section-level samples from the eight blades were retained in the final dataset used for the grouped evaluation. No samples were excluded based on CMM measurements or profile registration. During data acquisition, force and moment channels were monitored to identify any abnormal-contact events; however, all measured sections were included in the dataset regardless of transient force fluctuations. The resulting section-level dataset thus reflects the complete set of acquired measurements and provides a consistent basis for descriptor construction and subsequent predictive modeling.

The resulting section-level regression datasets are written as

(55) $$D_{\sec}^{\left(M\right)}=\left\{\left(x_{i}^{\left(M\right)},\;y_{i}\right)\;|\;i=1,2,\ldots ,80\right\},\;M\in \left\{A,B1,B2,B3\right\}$$

where $x_{i}^{\left(M\right)}$ denotes the section-level descriptor vector of the *i*th sample under model configuration *M*.

### 4.4. Performance Metrics

The grouped training and prediction protocol is defined in Section 3.3. This subsection specifies the metrics used to evaluate the held-out prediction records. Let $N_{t}=80$ denote the number of pooled held-out section-level predictions. For each model *M*, $y_{i}$ denotes the measured section-level RMS residual profile error and ${\hat{y}}_{i}^{\left(M\right)}$ denotes the corresponding point prediction. For GPR models, ${\hat{y}}_{i}^{\left(M\right)}={\hat{\mu }}_{y,i}^{\left(M\right)}$.

The deterministic prediction accuracy is evaluated using the root mean square error (RMSE), mean absolute error (MAE), mean bias error (MBE), maximum absolute error (MaxAE), and coefficient of determination ($R^{2}$):

(56) $${\mathrm{RMSE}}^{\left(M\right)}={\left[\frac{1}{N_{t}}\sum_{i=1}^{N_{t}}{\left({\hat{y}}_{i}^{\left(M\right)}-y_{i}\right)}^{2}\right]}^{1/2}$$

(57) $${\mathrm{MAE}}^{\left(M\right)}=\frac{1}{N_{t}}\sum_{i=1}^{N_{t}}\left|{\hat{y}}_{i}^{\left(M\right)}-y_{i}\right|$$

(58) $${\mathrm{MBE}}^{\left(M\right)}=\frac{1}{N_{t}}\sum_{i=1}^{N_{t}}\left({\hat{y}}_{i}^{\left(M\right)}-y_{i}\right)$$

(59) $${\mathrm{MaxAE}}^{\left(M\right)}=\max_{1\leq i\leq N_{t}}\left|{\hat{y}}_{i}^{\left(M\right)}-y_{i}\right|$$

(60) $$R^{2,(M)}=1-\frac{\sum_{i=1}^{N_{t}}{\left({\hat{y}}_{i}^{\left(M\right)}-y_{i}\right)}^{2}}{\sum_{i=1}^{N_{t}}{\left(y_{i}-\bar{y}\right)}^{2}}$$

where $\bar{y}=N_{t}^{-1}\sum_{i=1}^{N_{t}}y_{i}$ is the mean measured response of the pooled held-out sections. These deterministic metrics are computed for the training-mean predictor, Ridge-B3, and all GPR configurations.

For the GPR models, probabilistic performance is further evaluated using interval-based and distribution-based metrics. Let

(61) $$L_{i}^{\left(M\right)}={\hat{\mu }}_{y,i}^{\left(M\right)}-1.96{\hat{\sigma }}_{y,i}^{\left(M\right)}\;U_{i}^{\left(M\right)}={\hat{\mu }}_{y,i}^{\left(M\right)}+1.96{\hat{\sigma }}_{y,i}^{\left(M\right)}$$

denote the lower and upper bounds of the nominal 95% predictive interval. The empirical coverage is defined as

(62) $${\mathrm{Coverage}}_{95\%}^{\left(M\right)}=\frac{1}{N_{t}}\sum_{i=1}^{N_{t}}I\left(y_{i}\in \left[L_{i}^{\left(M\right)},U_{i}^{\left(M\right)}\right]\right)$$

where I(·) is the indicator function. The mean prediction interval width is

(63) $${\mathrm{MPIW}}_{95\%}^{\left(M\right)}=\frac{1}{N_{t}}\sum_{i=1}^{N_{t}}\left(U_{i}^{\left(M\right)}-L_{i}^{\left(M\right)}\right)$$

To jointly assess interval sharpness and missed observations, the interval score at nominal level 1−α=0.95 is computed as

(64) $${\mathrm{IS}}_{95\%}^{\left(M\right)}=\frac{1}{N_{t}}\sum_{i=1}^{N_{t}}\left[\left(U_{i}^{\left(M\right)}-L_{i}^{\left(M\right)}\right)+\frac{2}{\alpha }\left(L_{i}^{\left(M\right)}-y_{i}\right)I\left(y_{i}<L_{i}^{\left(M\right)}\right)+\frac{2}{\alpha }\left(y_{i}-U_{i}^{\left(M\right)}\right)I\left(y_{i}>U_{i}^{\left(M\right)}\right)\right]$$

For the Gaussian predictive distribution, the negative log predictive density is

(65) $${\mathrm{NLPD}}^{\left(M\right)}=\frac{1}{N_{t}}\sum_{i=1}^{N_{t}}\left[\frac{1}{2}\log\left(2\pi {\left({\hat{\sigma }}_{y,i}^{\left(M\right)}\right)}^{2}\right)+\frac{{\left(y_{i}-{\hat{\mu }}_{y,i}^{\left(M\right)}\right)}^{2}}{2{\left({\hat{\sigma }}_{y,i}^{\left(M\right)}\right)}^{2}}\right]$$

and the continuous ranked probability score is

(66) $${\mathrm{CRPS}}^{\left(M\right)}=\frac{1}{N_{t}}\sum_{i=1}^{N_{t}}{\hat{\sigma }}_{y,i}^{\left(M\right)}\left[z_{i}^{\left(M\right)}\left(2\Phi \left(z_{i}^{\left(M\right)}\right)-1\right)+2\varphi \left(z_{i}^{\left(M\right)}\right)-\frac{1}{\sqrt{\pi }}\right],\;z_{i}^{\left(M\right)}=\frac{y_{i}-{\hat{\mu }}_{y,i}^{\left(M\right)}}{{\hat{\sigma }}_{y,i}^{\left(M\right)}}$$

where Φ(·) and φ(·) denote the standard normal cumulative distribution function and probability density function, respectively. These probabilistic metrics are computed only for the GPR configurations.

Because the blade is the independent experimental unit, fold-wise metrics are also computed at the held-out-blade level. For the *r*th held-out blade and Model *M*, the fold-wise RMSE and MAE are

(67) $${\mathrm{RMSE}}_{r}^{\left(M\right)}={\left[\frac{1}{|I_{\mathrm{te}}^{\left(r\right)}|}\sum_{i\in I_{\mathrm{te}}^{\left(r\right)}}{\left({\hat{y}}_{i}^{\left(M\right)}-y_{i}\right)}^{2}\right]}^{1/2}$$

(68) $${\mathrm{MAE}}_{r}^{\left(M\right)}=\frac{1}{|I_{\mathrm{te}}^{\left(r\right)}|}\sum_{i\in I_{\mathrm{te}}^{\left(r\right)}}\left|{\hat{y}}_{i}^{\left(M\right)}-y_{i}\right|$$

Within the GPR family, the paired fold-wise improvements in GPR-B3 over GPR-A are defined as

(69) $$\Delta {\mathrm{RMSE}}_{r}={\mathrm{RMSE}}_{r}^{\left(A\right)}-{\mathrm{RMSE}}_{r}^{\left(B3\right)}\;\Delta {\mathrm{MAE}}_{r}={\mathrm{MAE}}_{r}^{\left(A\right)}-{\mathrm{MAE}}_{r}^{\left(B3\right)}$$

Since only eight independent blade-level folds are available, fold-wise differences are interpreted as descriptive paired trends rather than confirmatory statistical evidence. The pooled metrics characterize section-level prediction performance, whereas the fold-wise metrics characterize blade-to-blade variation in generalization performance.

## 5. Results and Discussion

Under the blade-wise grouped evaluation protocol, the four GPR configurations, the training-mean predictor, and the Ridge-B3 deterministic baseline were evaluated using the 80 pooled held-out section-level samples. Model A is the non-coupled GPR baseline, whereas Models B1, B2, and B3 progressively introduce the coupled descriptors $\phi_{1}$, $\phi_{2}$, and $\phi_{3}$. Ridge-B3 uses the same full descriptor set as GPR-B3 but assumes a linear mapping with $L_{2}$ regularization. The comparison is therefore interpreted at two levels: the predictive value of the sensor-derived mechanism-informed descriptors and the additional role of GPR as a probabilistic and interpretable model. All 80 section-level samples were retained in the analysis; no duplicated blade-edge-section keys or excluded outliers were identified in the descriptor dataset.

### 5.1. Comparative Deterministic Prediction Performance

Table 5 summarizes the deterministic prediction performance of all tested models. The training-mean predictor provides the lower-bound reference because it does not use any process, geometry, force-sensing, or metrology-derived descriptor. Its RMSE and MAE are 2.2319 μm and 1.7426 μm, respectively, and the negative $R^{2}$ indicates that the blade-wise held-out response cannot be predicted reliably by using only the average residual-error level of the training blades.

In contrast, Ridge-B3 reduces RMSE to 1.0285 μm and MAE to 0.8252 μm, with $R^{2}=0.7759$. This result indicates that the proposed sensor-derived and geometry-related descriptors contain strong predictive information for section-level residual profile error. The improvement from the training-mean predictor to Ridge-B3 is much larger than the incremental difference among the GPR configurations, showing that descriptor construction is the dominant source of deterministic prediction capability in the present dataset.

**Figure 7:**
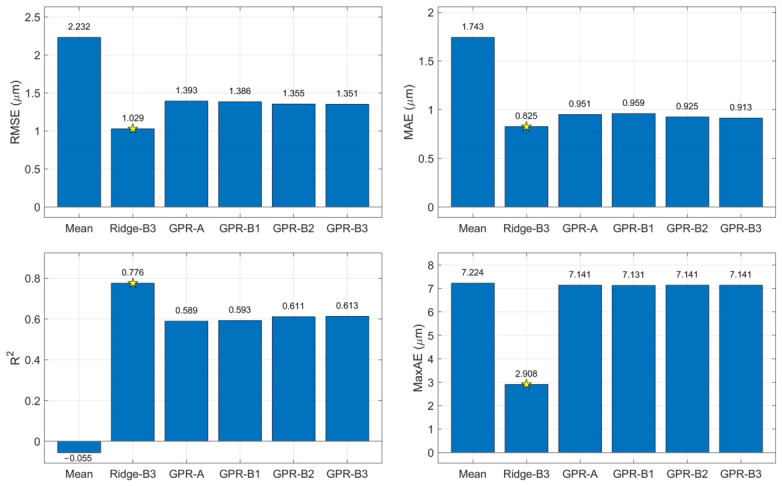
Deterministic prediction metrics of all tested models under the blade-wise grouped leave-one-blade-out protocol.

The same comparison is visualized in Figure 7. The yellow star marks the best-performing model for each metric. The RMSE, MAE, and MaxAE panels use non-negative error ranges, whereas the $R^{2}$ panel includes a small negative interval to retain the training-mean predictor. Under the pooled held-out definition in Equation (60), the negative $R^{2}$ value of the training-mean predictor indicates that its fold-wise mean predictions produce a larger total squared error than the pooled held-out mean reference. This result further shows that the section-level residual response cannot be represented by the average response of the training blades alone. Ridge-B3 gives the best deterministic performance across all four reported deterministic metrics. Among the GPR configurations, the full B3 descriptor set gives a modest improvement over GPR-A, but the differences within the GPR family remain much smaller than the gap between the descriptor-free training-mean predictor and the B3 descriptor-based models.

Within the GPR family, GPR-B3 reduces RMSE from 1.3926 μm to 1.3512 μm and MAE from 0.9508 μm to 0.9130 μm relative to GPR-A, corresponding to relative reductions of 2.97% and 3.98%, respectively. The $R^{2}$ value increases from 0.5892 to 0.6133. These results suggest that $\left\{\phi_{1},\phi_{2},\phi_{3}\right\}$ provide additional physically interpretable information on contact-load and radius-mismatch interactions, but the magnitude of this gain within the predefined Matérn-5/2 GPR family is limited.

Because the independent experimental unit is the physical blade rather than the individual section, the GPR-A to GPR-B3 difference was also evaluated at the blade-fold level. Table 6 reports the paired summary statistics over the eight held-out blade folds. The Wilcoxon signed-rank tests give p=0.4609 for RMSE and p=0.7422 for MAE, and the blade-level paired bootstrap intervals include zero for both metrics. Therefore, the improvement in GPR-B3 over GPR-A should be interpreted as an observed modest descriptor-induced trend rather than as a statistically established improvement.

**Figure 8:**
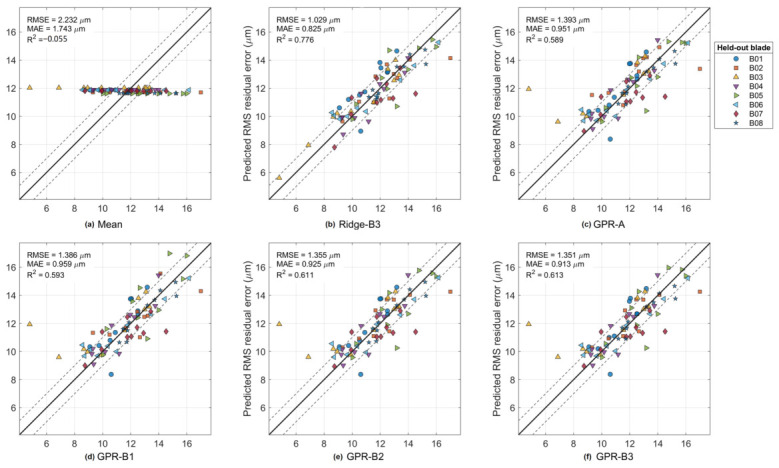
Parity plots of pooled held-out predictions for all tested models.

Figure 8 compares the measured and predicted section-level RMS residual profile errors for all six models. The training-mean predictor collapses the predictions toward the training-fold average and therefore cannot reproduce the measured response trend. Ridge-B3 provides the tightest deterministic clustering around the reference line and avoids the large GPR residual observed for one extrapolative leading-edge section. The GPR models capture the dominant monotonic trend, and GPR-B3 is slightly closer to the measured response than GPR-A, but the parity plots confirm the same conclusion as Table 5: the main deterministic advantage comes from the descriptor set, while the predefined GPR-B3 model should be viewed primarily as an uncertainty-aware extension.

The full per-blade fold-wise errors are reported in Supplementary Table S3 and visualized in Supplementary Figure S2. These fold-wise diagnostics support the use of conservative language for the GPR-B3 improvement because the eight blade folds provide limited degrees of freedom for formal statistical testing.

### 5.2. GPR Uncertainty and Calibration

Only the GPR-based models provide predictive distributions and nominal prediction intervals. Table 7 reports empirical coverage, interval sharpness, interval score, NLPD, CRPS, and the variance scaling factor required to reach 95% empirical coverage. GPR-A, GPR-B2, and GPR-B3 cover 73 of the 80 held-out sections under the nominal 95% predictive interval, corresponding to a coverage of 0.9125, whereas GPR-B1 covers 69 sections. Thus, the nominal intervals capture most observed variations but are mildly under-calibrated relative to the nominal 0.95 level.

GPR-B3 gives the lowest NLPD (1.6322) and CRPS (0.6796 μm), whereas GPR-B2 gives a slightly lower interval score (7.3471 versus 7.3662). The variance scaling factor required to reach 95% empirical coverage is 1.4185 for GPR-A, GPR-B2, and GPR-B3, corresponding to a standard deviation scaling factor of approximately 1.19. The predictive intervals should therefore be regarded as useful risk indicators for ranking uncertain sections rather than as complete metrological uncertainty bounds. This interpretation is also required because repeated CMM measurement and repeated registration uncertainty were not quantified for all 80 sections, so measurement and registration variations are included implicitly in the GPR observation-noise term.

**Figure 9:**
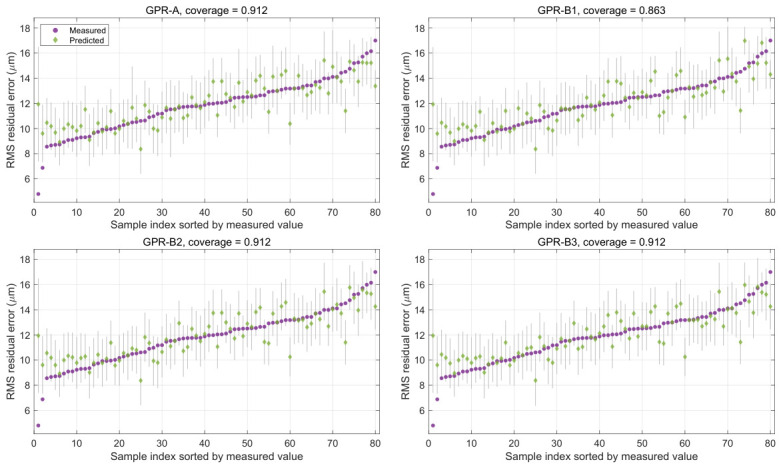
Pooled held-out predictions and nominal 95% predictive intervals for GPR-A, GPR-B1, GPR-B2, and GPR-B3.

Figure 9 visualizes the nominal 95% predictive intervals for all GPR configurations. The four GPR models show similar mean-response trends, and the B2/B3 configurations provide slightly improved probabilistic scores relative to GPR-A. However, several held-out sections remain outside the nominal intervals, which is consistent with the empirical under-coverage reported in Table 7.

### 5.3. Hyperparameter Behavior and Kernel Sensitivity

**Figure 10:**
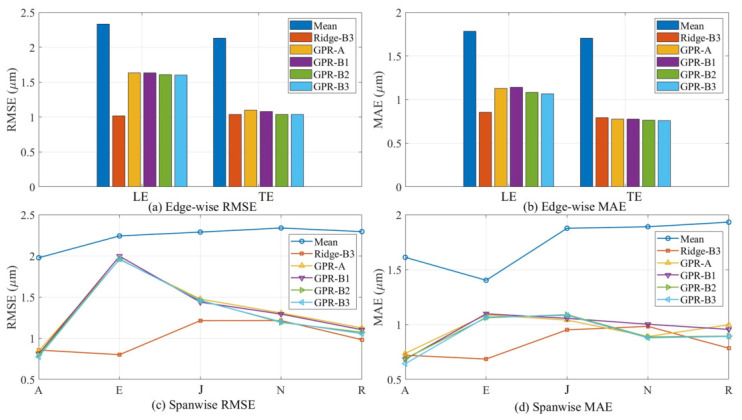
Edge- and spanwise-resolved deterministic prediction errors under the blade-wise grouped leave-one-blade-out protocol.

The pooled metrics were further decomposed by edge type and spanwise section to examine whether the prediction errors are spatially localized. Figure 10 shows the edge-wise and spanwise-resolved RMSE and MAE values for all tested models.

Panels (a) and (b) show edge-wise RMSE and MAE. Panels (c) and (d) show spanwise RMSE and MAE across sections A, E, J, N, and R. The training-mean predictor gives consistently large errors for both edge types and all spanwise sections, whereas the descriptor-based models substantially reduce the error. Ridge-B3 shows the most balanced deterministic performance across leading-edge and trailing-edge sections. The Matérn-5/2 GPR models exhibit larger errors on the leading-edge sections than on the trailing-edge sections, and their spanwise errors are highest at section E. This localized error amplification is consistent with the larger maximum absolute errors of the GPR models reported in Table 5. Therefore, the edge/spanwise analysis indicates that the proposed descriptors remain informative across the measured blade-edge regions, but the nonlinear GPR configurations are more sensitive to localized difficult sections than the regularized linear Ridge-B3 baseline.

The deterministic advantage of Ridge-B3 over the predefined Matérn-5/2 GPR-B3 can arise from an approximately linear descriptor–response relationship over the tested range, insufficient effective blade-level sample size for nonlinear GPR, or kernel/hyperparameter sensitivity. Table 8 summarizes representative GPR-B3 hyperparameters across the eight blade-wise folds. The optimized length scales are very large for most descriptor directions, whereas $\ell_{E_{0}}$ has a much smaller median value of 1.6093. This pattern indicates that the fitted Matérn-5/2 GPR-B3 model learns a relatively smooth response over most process and geometry descriptors and relies most strongly on the incoming profile-state descriptor $E_{0}$.

Kernel sensitivity was evaluated using the same B3 descriptor set and blade-wise grouped protocol. As shown in Table 9, the rational-quadratic kernel achieves RMSE = 1.0289 μm, MAE = 0.8226 μm, $R^{2}=0.7757$, and empirical 95% coverage of 0.9500. This performance is close to Ridge-B3 and substantially better than the predefined Matérn-5/2 GPR-B3. However, because the Matérn-5/2 ARD configuration was predefined in the main analysis, the rational-quadratic result is reported as kernel-sensitivity evidence rather than as test-set-based model selection. Thus, the rational-quadratic result is retained as kernel-sensitivity evidence and does not replace the predefined Matérn-5/2 configuration in the main comparison. Kernel selection under nested grouped validation will be considered in future extensions.

The kernel-sensitivity results in Table 9 show that the covariance-function choice affects both deterministic accuracy and uncertainty calibration. The rational-quadratic kernel gives the best result among the tested GPR-B3 variants, with RMSE = 1.0289 μm, MAE = 0.8226 μm, $R^{2}=0.7757$, and empirical 95% coverage of 0.9500. This performance suggests that a multi-scale covariance structure may better match the response variation in the B3 descriptor space than the predefined Matérn-5/2 ARD kernel. In the present study, however, the main GPR comparison is based on the fixed Matérn-5/2 ARD configuration defined before the grouped evaluation, whereas the rational-quadratic result is used to assess kernel sensitivity. Future work should incorporate kernel-family selection into a nested grouped cross-validation framework, with the inner blade-wise loop used for kernel and hyperparameter selection and the outer blade-wise split used only for final generalization assessment. Given the current dataset of eight independent blades, this extension requires a larger blade-level dataset to obtain stable kernel-selection results.

### 5.4. Deployment-Related Ablations and Error Diagnostics

The incoming profile-state descriptor $E_{0}$ is physically meaningful because it represents the realized pre-finishing sectional error, but it requires pre-finishing metrology or a validated in-process proxy. Table 10 reports the ablation result obtained after removing $E_{0}$ while retaining the same blade-wise grouped protocol. Without $E_{0}$, Ridge-B3 degrades from RMSE = 1.0285 μm to 2.0012 μm, and GPR-B3 degrades from RMSE = 1.3512 μm to 2.2531 μm. The current predictor is therefore best suited to inspection-assisted finishing or closed-loop compensation workflows where the incoming profile state is available.

Out-of-training-range diagnostics further explain the largest GPR-B3 residual and the mild under-coverage of nominal intervals. For GPR-B3, 21 out-of-training-range feature events were observed, affecting 16 held-out sections. The largest normalized extrapolation distance was 0.4172 and occurred for the $E_{0}$ descriptor of sample B03_LE_E, where $E_{0}=7.19\;\mu$m was below the corresponding training-fold minimum of 10.19 μm. The same sample produced the largest GPR-B3 absolute error of 7.1408 μm. Detailed out-of-training-range events, edge-wise errors, spanwise-position errors, pass/monitoring ablations, and residual boxplots are reported in Supplementary Tables S4, S5 and S8–S10 and Supplementary Figures S1, S4 and S5.

### 5.5. Descriptor Attribution in the Fitted GPR Models

To examine how the fitted probabilistic models use the constructed section-level descriptors, a model-agnostic SHAP analysis was applied to the predictive mean functions of GPR-A and GPR-B3. The analysis provides a global attribution view of the fitted mappings and complements the held-out prediction results reported above.

For a section-level descriptor vector x, the predictive mean can be expressed as

(70) $$\hat{y}\left(x\right)=\varphi_{0}+\sum_{k=1}^{d}\varphi_{k}\left(x\right),$$

where $\varphi_{0}$ denotes the expected model output over the background dataset and $\varphi_{k}\left(x\right)$ denotes the SHAP contribution of the *k*th descriptor. The mean absolute SHAP value over all analyzed sections is used to rank the global contribution of each descriptor.

**Figure 11:**
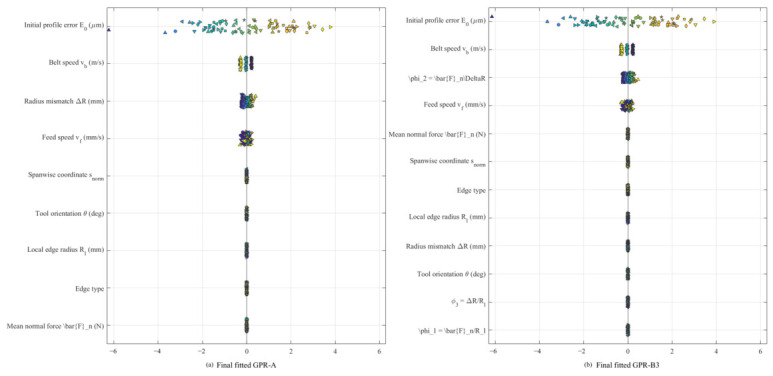
SHAP summary plots of the fitted GPR-A and GPR-B3 models.

Figure 11 shows that the initial profile-state descriptor $E_{0}$ is the dominant contributor in both GPR-A and GPR-B3. Its SHAP values span a much wider range than those of the remaining descriptors, indicating that the fitted predictive mean is primarily organized by the incoming sectional profile state. Sections with larger $E_{0}$ generally contribute positively to the predicted residual error, whereas sections with smaller $E_{0}$ contribute negatively. This trend is physically consistent with the corrective nature of the finishing process: the final residual profile error is not determined by nominal process parameters alone but remains strongly associated with the realized incoming profile state before finishing.

After the coupled descriptors are introduced in GPR-B3, the overall attribution structure remains dominated by $E_{0}$. Among the added interaction descriptors, $\phi_{2}={\bar{F}}_{n}\Delta R$ shows the most visible secondary contribution, while $\phi_{1}={\bar{F}}_{n}/R_{l}$ and $\phi_{3}=\Delta R/R_{l}$ remain close to zero for most sections. This indicates that the force–radius-mismatch interaction provides supplementary information beyond the basic descriptor set, whereas the load-to-radius ratio and normalized radius mismatch contribute weakly in the fitted GPR-B3 model.

Features are sorted by their mean absolute SHAP values within each model. The horizontal coordinate represents the descriptor contribution to the predicted section-level RMS residual profile error. Each point corresponds to one measured section, and marker shapes denote blade identity. Point color denotes the normalized value of the corresponding descriptor, with cooler colors indicating lower descriptor values and warmer colors indicating higher descriptor values.

The attribution pattern is consistent with the predictive results in Table 5. The coupled descriptors encode load–geometry and radius-mismatch interactions, but their contribution remains secondary to that of $E_{0}$, which is consistent with the modest deterministic change from GPR-A to GPR-B3. The interpretation of these attribution results is further constrained by the correlation structure of the B3 descriptor space. As shown by the collinearity diagnostics in Supplementary Table S11, several descriptors exhibit severe multicollinearity, with variance inflation factors exceeding 100. This behavior is expected because $\phi_{1}={\bar{F}}_{n}/R_{l}$, $\phi_{2}={\bar{F}}_{n}\Delta R$, and $\phi_{3}=\Delta R/R_{l}$ are algebraic combinations of the original load and geometry variables. The B3 descriptor set should therefore be viewed as a physically motivated coupled descriptor block rather than as a collection of mutually independent physical factors.

This correlation structure mainly affects the interpretation of model-internal quantities. For Ridge-B3, the $L_{2}$ penalty reduces the variance of the fitted linear mapping and supports stable held-out prediction, but it does not make the individual coefficients uniquely identifiable under strong descriptor redundancy. Correlated descriptors can share the same response variation, so the coefficient assigned to one descriptor may depend on the blade-wise fold composition and the selected regularization strength. Accordingly, the sign and magnitude of an individual Ridge-B3 coefficient are interpreted as part of a regularized descriptor-block representation, rather than as an independent marginal physical effect of a single process or geometry variable.

The same consideration applies to the ARD length scales in GPR-B3. ARD length scales describe the variation in the fitted covariance function along normalized input coordinates; however, under severe collinearity, these coordinate directions do not correspond to independent physical directions. The marginal likelihood may therefore allocate sensitivity non-uniquely within a correlated descriptor group, producing a relatively short or moderate length scale for one descriptor while assigning very large or unstable length scales to nearly redundant descriptors. The large length scales observed for several GPR-B3 descriptors are therefore interpreted as diagnostics of the fitted covariance structure and possible redundancy in the descriptor space, rather than as definitive evidence of physical irrelevance. Overall, SHAP values, Ridge-B3 coefficients, and GPR ARD length scales are used as model-specific diagnostic quantities for descriptor usage, while physical interpretation is made at the level of descriptor groups and the underlying finishing mechanism.

### 5.6. Limitations and Deployment Implications

Several limitations should be considered when interpreting these results. First, the independent experimental unit is the physical blade, so the effective sample size for between-blade generalization is eight rather than 80. Second, all blades were made of 2Cr13 stainless steel and finished on the same robot platform with the same abrasive-belt and contact-wheel specifications; cross-material, cross-machine, and cross-tool generalization remain unverified. Third, pass-state and monitoring summaries did not improve grouped held-out performance in the current 80-section dataset, but the absence of direct wheel-wear and wheel-compliance measurements remains a deployment limitation. Fourth, the GPR intervals are nominal and mildly under-calibrated, so they should be used as risk indicators rather than as a complete metrological uncertainty budget. Finally, the model predicts section-level RMS residual error rather than the signed pointwise deviation field. Extension toward pointwise profile-error prediction and closed-loop dwell-time compensation will require dense-profile modeling, explicit metrological uncertainty analysis, and validation on additional blades.

## 6. Conclusions

This study developed a sensor-derived mechanism-informed descriptor and prediction framework for section-level RMS residual profile error in robotic turbine-blade-edge finishing. Online force sensing, robot/process records, CAD-derived geometry, and CMM-based sectional metrology were converted into physically interpretable descriptors. Four GPR configurations, a training-mean predictor, and a Ridge-B3 baseline were evaluated under a blade-wise grouped leave-one-blade-out protocol. The main conclusions are summarized as follows:

1. The proposed descriptor system provides an effective representation of section-level blade-edge residual error. It links the response to normal force, feed speed, belt speed, tool orientation, local edge radius, radius mismatch, incoming profile state, edge type, spanwise location, and the coupled descriptors $\phi_{1}={\bar{F}}_{n}/R_{l}$, $\phi_{2}={\bar{F}}_{n}\Delta R$, and $\phi_{3}=\Delta R/R_{l}$. The poor performance of the training-mean predictor, with RMSE = 2.2319 μm, MAE = 1.7426 μm, and negative $R^{2}$, confirms that the residual response cannot be represented by the average training response alone.
2. Descriptor construction is the dominant source of deterministic prediction accuracy. Ridge-B3 achieved the best deterministic performance among the tested main models, with RMSE = 1.0285 μm, MAE = 0.8252 μm, MBE = 0.0478 μm, $R^{2}=0.7759$, and MaxAE = 2.9076 μm. This result shows that the proposed sensor-derived and geometry-related descriptors contain strong predictive information. In contrast, GPR-B3 did not outperform Ridge-B3 deterministically, indicating that the role of GPR should be interpreted mainly as uncertainty-aware modeling rather than as the strongest point predictor.
3. Within the predefined Matérn-5/2 GPR family, the coupled descriptors produced a modest improvement. Compared with GPR-A, GPR-B3 reduced RMSE from 1.3926 μm to 1.3512 μm and MAE from 0.9508 μm to 0.9130 μm, corresponding to reductions of 2.97% and 3.98%, respectively. However, the blade-level paired tests did not establish this improvement as statistically significant, with Wilcoxon p=0.4609 for RMSE and p=0.7422 for MAE. Therefore, the coupled descriptors should be interpreted as physically meaningful supplementary descriptors rather than as producing a strong deterministic gain in the current eight-blade dataset.
4. GPR provides useful but approximate uncertainty and attribution information. For GPR-B3, the empirical coverage of the nominal 95% predictive interval was 0.9125, with 73 of 80 held-out sections covered, indicating mild under-calibration. The SHAP analysis identified the incoming profile descriptor $E_{0}$ as the dominant contributor, while $\phi_{2}={\bar{F}}_{n}\Delta R$ provided a visible but secondary contribution and $\phi_{1}$ and $\phi_{3}$ remained weak for most sections. The strong degradation of both Ridge-B3 and GPR-B3 after removing $E_{0}$ further indicates that the current framework is most suitable for inspection-assisted finishing or closed-loop compensation workflows where the incoming profile state is available.

Overall, this study demonstrates that heterogeneous force, process, geometry, and metrology information can be converted into interpretable section-level descriptors for robotic blade-edge quality prediction. The results also clarify the respective roles of the tested model classes: ridge regression validates the deterministic predictive value of the descriptor set, whereas GPR provides an uncertainty-aware layer for risk-oriented assessment. Future work will expand the blade-level dataset, improve predictive-interval calibration, quantify CMM and registration uncertainty, develop reduced models or in-process proxies for E_0_, and extend the current section-level RMS predictor toward signed pointwise profile-deviation prediction and closed-loop dwell-time compensation.

## Figures and Tables

**Table 1:** Descriptor configurations used in the section-level regression models.

Model	Descriptor Vector	Dimension
A	$x_{i}^{\left(A\right)}$	9
B1	$x_{i}^{\left(B1\right)}={\left[{\left(x_{i}^{\left(A\right)}\right)}^{⊤},\phi_{1,i}\right]}^{⊤}$	10
B2	$x_{i}^{\left(B2\right)}={\left[{\left(x_{i}^{\left(B1\right)}\right)}^{⊤},\phi_{2,i}\right]}^{⊤}$	11
B3	$x_{i}^{\left(B3\right)}={\left[{\left(x_{i}^{\left(B2\right)}\right)}^{⊤},\phi_{3,i}\right]}^{⊤}$	12

**Table 2:** Common process settings and blade-level varied conditions in the finishing experiments.

Item	Setting
Rough finishing station	Upper station
Rough finishing abrasive belt	240-grit silicon carbide abrasive belt
Semi-finishing and finishing station	Lower station
Semi-finishing and finishing abrasive belt	600-grit silicon carbide abrasive belt
Contact-wheel diameter	25 mm
Contact-wheel width	10 mm
Reserved stock before semi-finishing	Varied among blade experiments to form different incoming profile states
Other process settings	Maintained according to the prescribed process plan

**Table 3:** Measured incoming stock state before semi-finishing represented by $E_{0}$.

Blade ID	No. of Sections	$A_{b}^{\mathrm{meas}}={\bar{E}}_{0,b}$	Std. of $E_{0}$	Range of $E_{0}$
B01	10	13.38 μm	1.79 μm	10.19–15.81 μm
B02	10	14.40 μm	1.51 μm	12.39–16.35 μm
B03	10	12.72 μm	2.68 μm	7.19–15.75 μm
B04	10	13.46 μm	1.91 μm	11.20–17.10 μm
B05	10	14.63 μm	1.87 μm	11.59–17.19 μm
B06	10	13.34 μm	1.99 μm	11.49–17.38 μm
B07	10	13.43 μm	1.33 μm	10.54–15.15 μm
B08	10	14.40 μm	1.35 μm	12.43–16.41 μm
Overall	80	13.72 μm	1.88 μm	7.19–17.38 μm

**Table 4:** CMM repeatability and registration uncertainty check for selected blade-edge sections.

Item	No. of Sections	No. of Repeats	Mean Std. of RMS Error [μm]	Maximum Std. [μm]
Repeated CMM probing	5	3	0.35	0.48
Repeated registration	5	5	0.22	0.30
Combined check	5	3	0.42	0.50

**Table 5:** Comparison of deterministic prediction performance under the blade-wise grouped leave-one-blade-out protocol. Error metrics are reported in μm unless otherwise noted.

Model	*N*	RMSE	MAE	MBE	$R^{2}$	MaxAE
Training-mean predictor	80	2.2319	1.7426	0.0000	−0.0551	7.2240
Ridge-B3	80	1.0285	0.8252	0.0478	0.7759	2.9076
GPR-A	80	1.3926	0.9508	0.2061	0.5892	7.1408
GPR-B1	80	1.3858	0.9591	0.2320	0.5932	7.1305
GPR-B2	80	1.3551	0.9246	0.1748	0.6110	7.1408
GPR-B3	80	1.3512	0.9130	0.1694	0.6133	7.1408

**Table 6:** Blade-level paired comparison between GPR-A and GPR-B3. Positive improvement denotes lower error for GPR-B3.

Metric	Mean Improvement	Median Improvement	Wilcoxon *p*	Bootstrap 95% CI
RMSE	0.0413	0.0042	0.4609	[−0.0341, 0.1502]
MAE	0.0378	0.0031	0.7422	[−0.0302, 0.1326]

**Table 7:** Probabilistic performance of nominal GPR predictive distributions. Deterministic baselines are excluded because they do not output predictive intervals.

Metric	GPR-A	GPR-B1	GPR-B2	GPR-B3
Coverage of nominal 95% PI	0.9125	0.8625	0.9125	0.9125
Covered sections	73	69	73	73
Missed sections	7	11	7	7
Mean 95% PI width (μm)	3.9895	3.8477	3.8548	3.8596
Interval score, 95%	8.2323	8.8791	7.3471	7.3662
NLPD	1.7826	1.9082	1.6336	1.6322
CRPS (μm)	0.7047	0.7252	0.6828	0.6796
Variance scaling factor for 95% empirical coverage	1.4185	2.7307	1.4185	1.4185

**Table 8:** Compact summary of optimized GPR-B3 hyperparameters across eight blade-wise folds. The complete length-scale table is provided in Supplementary Table S6.

Hyperparameter or Length Scale	Median	Interquartile Range
$\sigma_{f}$	7.6386	10.5567
$\sigma_{n}$	0.9187	0.0981
$\ell_{E_{0}}$	1.6093	2.4767
$\ell_{\phi_{2}}$	19.9551	1870.8161
$\ell_{v_{b}}$	20.9971	15.5117
$\ell_{{\bar{F}}_{n}}$	$2.42\times 10^{5}$	$3.14\times 10^{6}$
$\ell_{R_{l}}$	$4.00\times 10^{5}$	$2.12\times 10^{6}$
$\ell_{\phi_{1}}$	$1.18\times 10^{6}$	$4.91\times 10^{10}$

**Table 9:** Kernel-sensitivity comparison for GPR-B3 under the same blade-wise grouped leave-one-blade-out protocol.

Kernel	RMSE	MAE	$R^{2}$	Coverage 95	CRPS
Matérn-5/2 ARD	1.3512	0.9130	0.6133	0.9125	0.6796
Matérn-3/2 ARD	1.3141	0.9124	0.6342	0.9125	0.6741
Squared-exponential ARD	1.3831	0.9153	0.5948	0.9000	0.6881
Rational-quadratic	1.0289	0.8226	0.7757	0.9500	0.5833

**Table 10:** Ablation analysis for the incoming profile-state descriptor $E_{0}$.

Model	Descriptor Setting	RMSE	MAE	$R^{2}$
Ridge-B3	Full B3 with $E_{0}$	1.0285	0.8252	0.7759
Ridge-B3-no-$E_{0}$	B3 excluding $E_{0}$	2.0012	1.5831	0.1517
GPR-B3	Full B3 with $E_{0}$	1.3512	0.9130	0.6133
GPR-B3-no-$E_{0}$	B3 excluding $E_{0}$	2.2531	1.7870	−0.0753

## Data Availability

The anonymized section-level descriptor dataset and the evaluation scripts used for the grouped leave-one-blade-out analysis are provided as Supplementary Materials. Raw CMM contours and CAD geometry are not publicly available due to industrial confidentiality but may be made available for peer-review verification upon reasonable request.

## Supplementary Materials

The following supporting information can be downloaded at: https://www.mdpi.com/article/10.3390/s26123799/s1, Supplementary Note S1: Additional probabilistic metrics; Supplementary Note S2: Dataset audit and descriptor distribution; Supplementary Note S3: Fold-wise model comparison; Supplementary Note S4: Out-of-training-range diagnostics; Supplementary Note S5: GPR-B3 hyperparameter stability; Supplementary Note S6: Kernel sensitivity; Supplementary Note S7: Edge-wise and spanwise error diagnostics; Supplementary Note S8: Descriptor-set ablations; Supplementary Note S9: Descriptor collinearity; Table S1: Dataset audit; Table S2: Summary statistics of section-level descriptors and response; Table S3: Fold-wise deterministic errors for GPR-A and GPR-B3; Table S4: Out-of-training-range feature-event summary under fold-wise min–max normalization; Table S5: Detailed out-of-training-range feature events for GPR-B3; Table S6: Complete summary of optimized GPR-B3 hyperparameters across eight blade-wise folds; Table S7: Full kernel-sensitivity results for the B3 descriptor set under the same blade-wise grouped protocol; Table S8: Prediction error by edge type for Ridge-B3 and GPR-B3; Table S9: GPR-B3 prediction error by spanwise section position; Table S10: Ablation and auxiliary-descriptor results under the blade-wise grouped protocol; Table S11: Variance inflation factors for B3 descriptors; Table S12: Representative Pearson correlations among important descriptors; Figure S1: Residual and absolute-error distributions of all tested models under the blade-wise grouped protocol; Figure S2: Fold-wise RMSE and MAE heatmaps for the six tested models; Figure S3: Probabilistic performance metrics of the four GPR configurations; Figure S4: Kernel-sensitivity comparison for the B3 descriptor set under the same blade-wise grouped protocol; Figure S5: Ablation metrics for $E_{0}$, pass-state, and monitoring-summary variables under the blade-wise grouped protocol.

## Abbreviations

The following abbreviations are used in this manuscript:

CMM	Coordinate measuring machine
CRPS	Continuous ranked probability score
GPR	Gaussian process regression
LOBO	Leave-one-blade-out
MAE	Mean absolute error
MaxAE	Maximum absolute error
MBE	Mean bias error
MPIW	Mean prediction interval width
NLPD	Negative log predictive density
PI	Prediction interval
PIT	Probability integral transform
RMSE	Root mean square error
RMS	Root mean square
SHAP	SHapley Additive exPlanations
SVR	Support vector regression
