# Supplementary material for: Sensor-Derived Mechanism-Informed Prediction of Section-Level Residual Profile Error in Robotic Blade-Edge Finishing
Source: Sensors (Basel). 2026 Jun 15;26(12):3799. doi: 10.3390/s26123799 (PMC13306834; doi:10.3390/s26123799)
Supplement: Supplementary file 1 [file sensors-26-03799-s001.zip › sensors-4334631-supplementary.pdf]

sensors

MDPI

Article

Sensor-Derived Mechanism-Informed Prediction of Section-Level Residual Profile Error in Robotic Blade-Edge Finishing

Zhuohang Gao, Xi Zeng *, Zhenyu Cai and Cong Wen

Supplementary Materials

Supplementary Note S1. Additional probabilistic metrics

For a nominal $(1-\alpha)$ predictive interval $[l_i, u_i]$ with predictive mean $\hat{\mu}_i$ and standard deviation $\hat{\sigma}_i$, the mean prediction interval width, interval score, negative log predictive density, and continuous ranked probability score were computed as diagnostic metrics:

$$\mathrm{MPIW} = \frac{1}{N}\sum_{i=1}^{N}(u_i - l_i), \tag{S1}$$

$$\mathrm{IS}_\alpha = \frac{1}{N}\sum_{i=1}^{N}\left[(u_i - l_i) + \frac{2}{\alpha}(l_i - y_i)\mathbb{I}(y_i < l_i) + \frac{2}{\alpha}(y_i - u_i)\mathbb{I}(y_i > u_i)\right], \tag{S2}$$

$$\mathrm{NLPD} = \frac{1}{N}\sum_{i=1}^{N}\left[\frac{1}{2}\log(2\pi\hat{\sigma}_i^2) + \frac{(y_i - \hat{\mu}_i)^2}{2\hat{\sigma}_i^2}\right]. \tag{S3}$$

For a Gaussian predictive distribution, CRPS was computed using the standard closed form

$$\mathrm{CRPS} = \frac{1}{N}\sum_{i=1}^{N}\hat{\sigma}_i\left[z_i(2\Phi(z_i) - 1) + 2\phi(z_i) - \frac{1}{\sqrt{\pi}}\right], \quad z_i = \frac{y_i - \hat{\mu}_i}{\hat{\sigma}_i}, \tag{S4}$$

where $\Phi(\cdot)$ and $\phi(\cdot)$ are the standard normal cumulative distribution and density functions. The variance scaling factor required for 95% empirical coverage was estimated from the ordered absolute standardized residuals $|y_i - \hat{\mu}_i|/\hat{\sigma}_i$.

Supplementary Note S2. Dataset audit and descriptor distribution

Table S1. Dataset audit.

Item	Value
Rows	80
Variables	25
Unique blades	8
Duplicate sample keys	0
Target mean	11.8048
Target std	2.1865
Target min	4.8000
Target max	17.0000
Pearson corr(E0,target)	0.8899

 https://doi.org/10.3390/s26123799

Table S2. Summary statistics of section-level descriptors and response.

Variable	N	Mean	Std.	Min	Median	Max
edge-type code	80	0.5000	0.5032	0.0000	0.5000	1.0000
s_{norm}	80	0.5000	0.2846	0.1000	0.5000	0.9000
R_l (mm)	80	0.3176	0.1499	0.0810	0.3115	0.5570
ΔR (mm)	80	0.0578	0.0376	0.0050	0.0590	0.1420
θ (deg)	80	15.3899	2.9279	9.2400	15.0250	21.0800
v_f (mm/s)	80	12.0000	3.4860	8.0000	12.0000	16.0000
v_b (m/s)	80	27.6250	2.3566	25.0000	28.0000	31.0000
$\bar{F}_n$ (N)	80	12.9409	1.0026	10.7500	12.9700	14.8500
E_0 (μm)	80	13.7210	1.8750	7.1900	13.7200	17.3800
$\phi_1 = \bar{F}_n / R_l$	80	54.8226	33.6493	20.6929	40.9681	156.5432
$\phi_2 = \bar{F}_n \Delta R$	80	0.7511	0.5027	0.0590	0.7367	1.9951
$\phi_3 = \Delta R / R_l$	80	0.2215	0.1776	0.0118	0.1941	0.9012
y (μm)	80	11.8048	2.1865	4.8000	11.9250	17.0000
pass ID	80	1.5000	0.5032	1.0000	1.5000	2.0000
F_n std. (N)	80	2.1046	0.1287	1.7900	2.1100	2.3900
F_n max. (N)	80	17.1737	1.2901	14.6000	17.1500	20.2200
mean moment (N m)	80	0.5885	0.0696	0.4130	0.5935	0.7480
contact time (s)	80	0.5477	0.0496	0.4570	0.5460	0.6460

Supplementary Note S3. Fold-wise model comparison

Table S3. Fold-wise deterministic errors for GPR-A and GPR-B3.

Held-out blade	RMSE GPR-A	RMSE GPR-B3	ΔRMSE	MAE GPR-A	MAE GPR-B3	ΔMAE
B01	1.2439	1.1966	0.0473	1.0268	0.9571	0.0696
B02	1.5685	1.1841	0.3845	1.2534	0.9176	0.3358
B03	2.5490	2.5490	-0.0000	1.5241	1.5241	-0.0000
B04	0.8535	0.8612	-0.0077	0.7268	0.7407	-0.0138
B05	1.1637	1.2811	-0.1174	0.9038	1.0104	-0.1066
B06	0.9241	0.9183	0.0058	0.7453	0.7391	0.0062
B07	1.3074	1.3049	0.0025	1.0156	1.0177	-0.0021
B08	0.6033	0.5881	0.0152	0.4110	0.3975	0.0134
Mean	1.2767	1.2354	0.0413	0.9508	0.9130	0.0378

Supplementary Note S4. Out-of-training-range diagnostics

Table S4. Out-of-training-range feature-event summary under fold-wise min–max normalization.

Model	Total events	Affected sections	Max distance	Most frequent predictors
GPR-A	15	14	0.4172	F_mean_N, theta_deg, R_nom_mm
GPR-B1	17	14	0.4172	F_mean_N, theta_deg, phi_load_over_R
GPR-B2	19	15	0.4172	F_mean_N, theta_deg, phi_load_over_R
GPR-B3	21	16	0.4172	F_mean_N, theta_deg, phi_load_over_R

https://doi.org/10.3390/s26123799

Table S5. Detailed out-of-training-range feature events for GPR-B3.

Held-out blade	Sample key	Predictor	Test value	Train min	Train max	Distance
B01	B01_TE_A	theta_deg	9.6200	9.9400	21.0800	0.0287
B01	B01_TE_E	theta_deg	9.2400	9.9400	21.0800	0.0628
B01	B01_LE_A	F_mean_N	11.0500	11.3200	14.8500	0.0765
B01	B01_LE_G	F_mean_N	10.7500	11.3200	14.8500	0.1615
B01	B01_LE_N	F_mean_N	11.3100	11.3200	14.8500	0.0028
B01	B01_TE_A	F_mean_N	11.1400	11.3200	14.8500	0.0510
B01	B01_TE_G	F_mean_N	10.8600	11.3200	14.8500	0.1303
B01	B01_TE_N	F_mean_N	11.2400	11.3200	14.8500	0.0227
B01	B01_LE_A	phi_load_over_R	20.6929	23.1239	156.5432	0.0182
B02	B02_LE_A	R_nom_mm	0.5570	0.0810	0.5490	0.0171
B03	B03_LE_A	delta_R_geom_mm	0.1420	0.0050	0.1370	0.0379
B03	B03_LE_E	e_pre_rms_um	7.1900	10.1900	17.3800	0.4172
B03	B03_LE_A	phi_load_times_dR	1.9951	0.0590	1.7899	0.1185
B04	B04_TE_R	R_nom_mm	0.0810	0.0930	0.5570	0.0259
B04	B04_LE_R	theta_deg	21.0800	9.2400	20.4800	0.0534
B04	B04_TE_R	phi_load_over_R	156.5432	20.6929	128.9247	0.2552
B04	B04_TE_N	phi_load_times_dR	0.0590	0.0637	1.9951	0.0024
B04	B04_TE_R	phi_dR_over_R	0.9012	0.0118	0.7742	0.1666
B06	B06_TE_N	e_pre_rms_um	17.3800	7.1900	17.1900	0.0190
B06	B06_LE_R	phi_dR_over_R	0.0118	0.0119	0.9012	0.0001
B08	B08_TE_A	F_mean_N	14.8500	10.7500	14.7500	0.0250

Supplementary Note S5. GPR-B3 hyperparameter stability

Table S6. Complete summary of optimized GPR-B3 hyperparameters across eight blade-wise folds.

Hyperparameter or descriptor length scale	Median	IQR	Q25	Q75
β_0	10.6650	0.6074	10.4987	11.1061
σ_f	7.6386	10.5567	3.6006	14.1573
σ_n	0.9187	0.0981	0.8686	0.9668
Log likelihood	-105.3738	3.7651	-107.9191	-104.1540
Negative log likelihood	105.3738	3.7651	104.1540	107.9191
ℓ_{b_e}	1.03×10^5	2.74×10^6	4.05×10^4	2.78×10^6
$\ell_{s_{\text{norm}}}$	1.75×10^5	1.56×10^7	2.72×10^4	1.56×10^7
ℓ_{R_l}	4×10^5	2.12×10^6	4.8×10^4	2.17×10^6
$\ell_{\Delta R}$	6612.3231	3.24×10^5	3593.4354	3.28×10^5
ℓ_θ	1.46×10^5	3.09×10^6	2.05×10^4	3.11×10^6
ℓ_{v_f}	6.0723	2.33×10^6	4.0677	2.33×10^6
ℓ_{v_b}	20.9971	15.5117	14.6487	30.1604
ℓ_{F_n}	2.42×10^5	3.14×10^6	8169.8858	3.15×10^6
ℓ_{E_0}	1.6093	2.4767	0.3828	2.8595
ℓ_{ϕ_1}	1.18×10^6	4.91×10^{10}	7.1×10^4	4.91×10^{10}
ℓ_{ϕ_2}	19.9551	1870.8161	8.5877	1879.4037
ℓ_{ϕ_3}	4.92×10^4	4.88×10^7	5366.2411	4.88×10^7

Supplementary Note S6. Kernel sensitivity

Table S7. Full kernel-sensitivity results for the B3 descriptor set under the same blade-wise grouped protocol.

Kernel	RMSE	MAE	MBE	R^2	MaxAE	Coverage95	MPIW95	IS95	NLPD	CRPS
Matérn-5/2 ARD	1.3512	0.9130	0.1694	0.6133	7.1408	0.9125	3.8596	7.3662	1.6322	0.6796
Matérn-3/2 ARD	1.3141	0.9124	0.1562	0.6342	6.5093	0.9125	3.8827	7.0659	1.6213	0.6741
Squared-exponential ARD	1.3831	0.9153	0.2052	0.5948	7.6611	0.9000	3.9542	7.6548	1.6841	0.6881
Rational-quadratic	1.0289	0.8226	0.0585	0.7757	2.9586	0.9500	4.2291	5.4447	1.4825	0.5833

Supplementary Note S7. Edge-wise and spanwise error diagnostics

Table S8. Prediction error by edge type for Ridge-B3 and GPR-B3.

Model	Edge	N	RMSE	MAE	MBE	MaxAE
Ridge-B3	LE	40	1.0171	0.8567	0.0476	2.4730
Ridge-B3	TE	40	1.0399	0.7937	0.0479	2.9076
GPR-B3	LE	40	1.6039	1.0660	0.2483	7.1408
GPR-B3	TE	40	1.0387	0.7600	0.0906	3.0950

Table S9. GPR-B3 prediction error by spanwise section position.

Section	s_{norm}	N	RMSE	MAE	MBE	MaxAE
A	0.1	16	0.7747	0.6432	0.0347	1.6018
E	0.3	16	1.9627	1.0682	0.5163	7.1408
J	0.5	16	1.4558	1.0834	0.2272	3.0950
N	0.7	16	1.2005	0.8789	-0.1075	2.7421
R	0.9	16	1.0562	0.8914	0.1765	1.8854

Supplementary Note S8. Descriptor-set ablations

Table S10. Ablation and auxiliary-descriptor results under the blade-wise grouped protocol.

Model	N	RMSE	MAE	MBE	R^2	MaxAE	Coverage95
Ridge-B3-no-E0	80	2.0012	1.5831	0.1458	0.1517	6.3897	–
GPR-B3-no-E0	80	2.2531	1.7870	-0.0602	-0.0753	6.7068	0.9250
Ridge-B3+pass	80	1.1047	0.8915	0.1358	0.7415	3.1919	–
GPR-B3+pass	80	1.3662	0.9333	0.1813	0.6046	7.1327	0.9125
Ridge-B3+monitoring	80	1.0554	0.8411	0.0407	0.7641	2.9142	–
GPR-B3+monitoring	80	1.3981	0.9576	0.1460	0.5860	7.1430	0.8875

Supplementary Note S9. Descriptor collinearity

Table S11. Variance inflation factors for B3 descriptors.

Predictor	VIF
$\phi_2 = \bar{F}_n \Delta R$	199.0051
ΔR (mm)	196.7943
R_l (mm)	123.4530
edge-type code	97.2380
s_{norm}	18.4761
$\phi_1 = \bar{F}_n / R_l$	10.9008
$\phi_3 = \Delta R / R_l$	8.7015
$\bar{F}_n$ (N)	6.7809
θ (deg)	5.2805
v_f (mm/s)	3.1366
E_0 (μm)	1.7643
v_b (m/s)	1.3206

Table S12. Representative Pearson correlations among important descriptors.

Descriptor pair	Correlation coefficient
$\phi_2 = \bar{F}_n \Delta R$ vs. ΔR (mm)	0.9904
$\phi_1 = \bar{F}_n / R_l$ vs. R_l (mm)	-0.8981
edge-type code vs. R_l (mm)	-0.9135
s_{norm} vs. θ (deg)	0.7746
$\bar{F}_n$ (N) vs. v_f (mm/s)	0.7646
$\phi_3 = \Delta R / R_l$ vs. ΔR (mm)	0.5145
E_0 vs. y	0.8899

https://doi.org/10.3390/s26123799

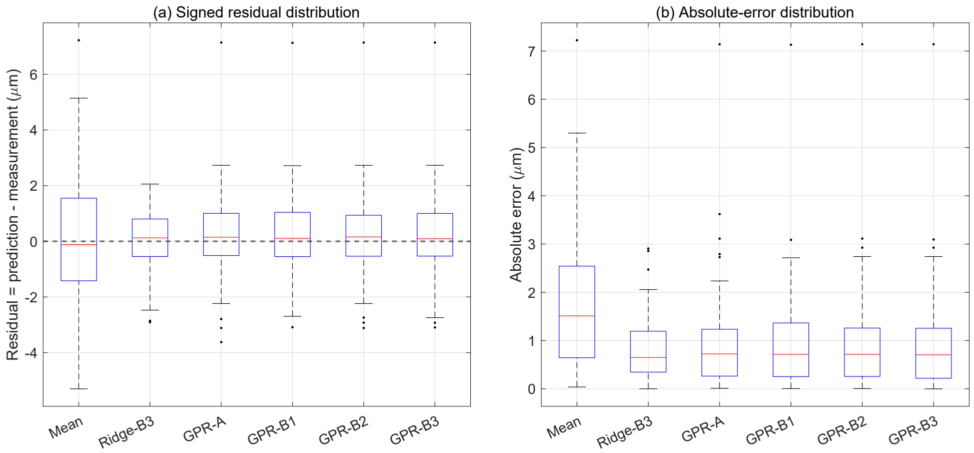

Figure S1. Residual and absolute-error distributions of all tested models under the blade-wise grouped protocol.

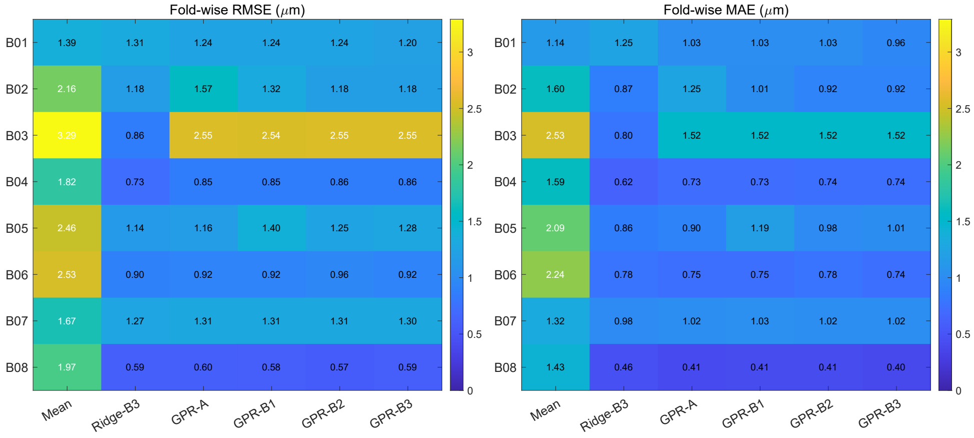

Figure S2. Fold-wise RMSE and MAE heatmaps for the six tested models. Each row corresponds to one held-out blade.

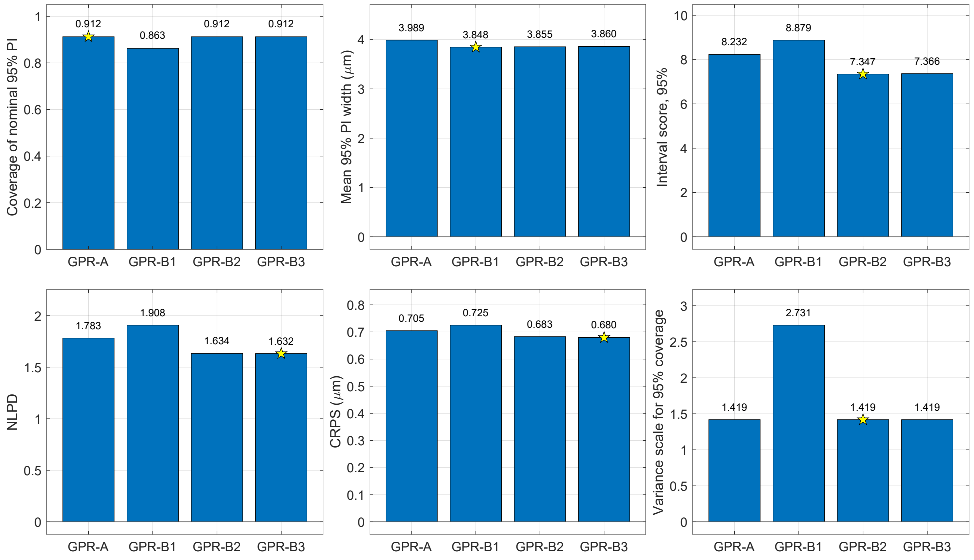

Figure S3. Probabilistic performance metrics of the four GPR configurations. Deterministic baselines are excluded because they do not output predictive distributions.

https://doi.org/10.3390/s26123799

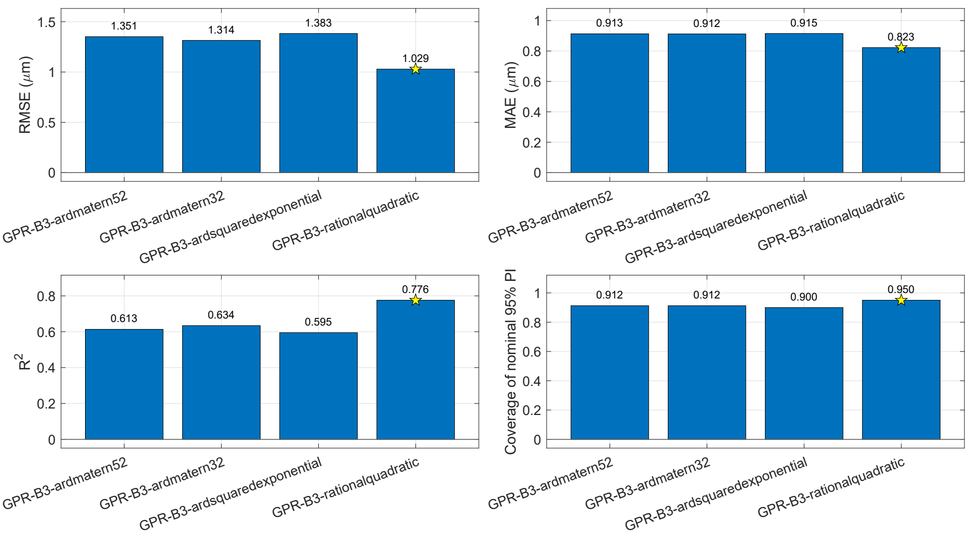

Figure S4. Kernel-sensitivity comparison for the B3 descriptor set under the same blade-wise grouped protocol.

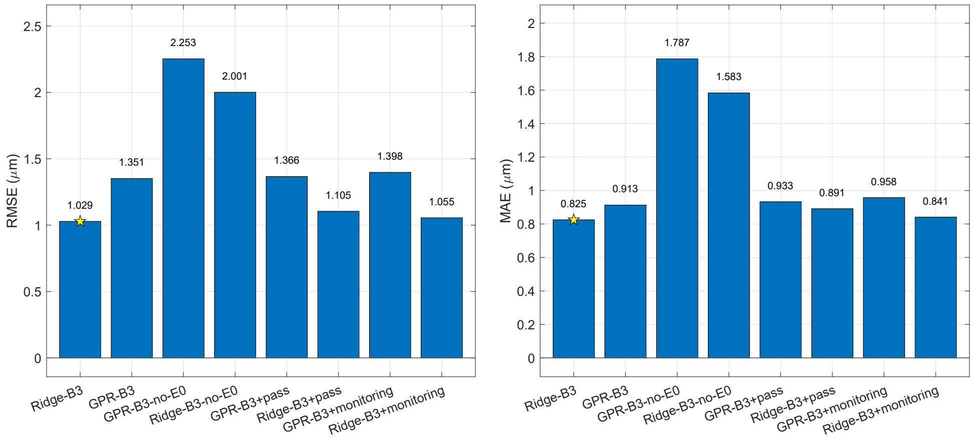

Figure S5. Ablation metrics for E_0, pass-state, and monitoring-summary variables under the blade-wise grouped protocol.
